# Comparison of linear and nonlinear implementation of the compartmental tissue uptake model for dynamic contrast‐enhanced MRI

**DOI:** 10.1002/mrm.26324

**Published:** 2016-09-08

**Authors:** Jesper F. Kallehauge, Steven Sourbron, Benjamin Irving, Kari Tanderup, Julia A. Schnabel, Michael A. Chappell

**Affiliations:** ^1^ Institute of Biomedical EngineeringDepartment of Engineering Science University of OxfordOxfordUnited Kingdom; ^2^ Division of Biomedical ImagingUniversity of LeedsLeedsUnited Kingdom; ^3^ Department of OncologyAarhus University HospitalAarhusDenmark; ^4^ Division of Imaging Science and Biomedical EngineeringKing's College LondonLondonUnited Kingdom.

**Keywords:** DCE‐MRI, tracer kinetic modeling, pharmacokinetics, cervical cancer, linear least‐squares method, nonlinear least‐squares

## Abstract

**Purpose:**

Fitting tracer kinetic models using linear methods is much faster than using their nonlinear counterparts, although this comes often at the expense of reduced accuracy and precision. The aim of this study was to derive and compare the performance of the linear compartmental tissue uptake (CTU) model with its nonlinear version with respect to their percentage error and precision.

**Theory and Methods:**

The linear and nonlinear CTU models were initially compared using simulations with varying noise and temporal sampling. Subsequently, the clinical applicability of the linear model was demonstrated on 14 patients with locally advanced cervical cancer examined with dynamic contrast‐enhanced magnetic resonance imaging.

**Results:**

Simulations revealed equal percentage error and precision when noise was within clinical achievable ranges (contrast‐to‐noise ratio >10). The linear method was significantly faster than the nonlinear method, with a minimum speedup of around 230 across all tested sampling rates. Clinical analysis revealed that parameters estimated using the linear and nonlinear CTU model were highly correlated (ρ ≥ 0.95).

**Conclusion:**

The linear CTU model is computationally more efficient and more stable against temporal downsampling, whereas the nonlinear method is more robust to variations in noise. The two methods may be used interchangeably within clinical achievable ranges of temporal sampling and noise. Magn Reson Med 77:2414–2423, 2017. © 2016 The Authors Magnetic Resonance in Medicine published by Wiley Periodicals, Inc. on behalf of International Society for Magnetic Resonance in Medicine. This is an open access article under the terms of the Creative Commons Attribution License, which permits use, distribution and reproduction in any medium, provided the original work is properly cited.

## INTRODUCTION

Dynamic contrast‐enhanced MRI (DCE‐MRI) is a powerful tool to evaluate tissue perfusion, permeability, and vasculature. High temporal resolution scans are performed while a gadolinium‐based contrast agent is introduced into the patient's blood stream, and its subsequent uptake is recorded. Numerous methods of analyzing the temporal tissue enhancement profile have been proposed, including phenomenological, semiquantitative, and quantitative tracer kinetic models 1, 2. The most commonly used tracer kinetic models are the Tofts and extended Tofts models, which have proven useful in a variety of clinical applications 3, 4. The limitations of the extended Tofts model have been revealed recently, as experimental evidence has shown that this model often fits poorly to data measured at high temporal resolution 5.

This has recently raised an increased interest in the use of more general models, such as the two‐compartment exchange model (2CXM) 6. The 2CXM allows for the description of two distinct compartments (v_e_ and v_p_) and separation of flow (F_p_) and the permeability surface area product (PS). For reliable estimation of all four parameters, a good contrast‐to‐noise ratio (CNR), high temporal resolution, and sufficiently long scan duration is required 7. The compartmental tissue uptake (CTU) model is a special case of the 2CXM which applies particularly for data with shorter scan durations 8, 9. This model has only three parameters (F_p_, PS, v_p_) and can be applied whenever the acquisition time is shorter than the contrast agent's extravascular transit times (typically in the range of 2–3 min, but significantly extended in some pathologies). The CTU model is a direct generalization of the well‐known Patlak model 10, which applies to data with poorer temporal resolution. An important application of the CTU model may be in tissues that include necrosis or cell membrane rupture where the contrast agent is captured for a long time. In those cases it is practically impossible to measure long enough to capture the washout phase.

Regardless of the choice of model for data analysis, the parameter estimation is often performed using a nonlinear fitting algorithm. Nonlinear methods require an initial guess of the parameters to be estimated and may converge only to a local minimum. Conversely, linear methods determine the kinetic parameters by solving a set of linear equations, as exemplified by Murase 11 and Flouri et al. 12 for DCE‐MRI, and there is also extensive experience in nuclear medicine 13. This is often much faster than the nonlinear approaches, as the optimum can be identified analytically by a single matrix inversion in a closed‐form solution, rather than iteratively via gradient‐descent type methods. The drawback often encountered with linear approaches is the higher sensitivity to noise and possible bias due to differences in data weighting 14. The nature and severity of the problem is model dependent, but these issues have not yet been investigated in the specific context of the CTU model.

The aim of this study was to formulate the CTU model in a linear form and compare the precision and percentage error of the parameter estimates of both linear and nonlinear solutions. The evaluation was performed using simulations with various noise levels and temporal downsampling. The applicability of the linear approach was demonstrated on 14 clinical cases of locally advanced cervical cancer.

## THEORY

The CTU model is a special case of the 2CXM, which is valid whenever the indicator concentration in the plasma volume (v_p_) is much larger than that in the extravascular distribution volume (v_e_) (i.e., c_p_ >> c_e_). It is governed by the following set of coupled linear differential equations:

(1) $$v_{p}\frac{dc_{p}(t)}{dt}=-PSc_{p}\left(t\right)+F_{p}(c_{a}\left(t\right)-c_{p}\left(t\right))$$

(2) $$v_{e}\frac{dc_{e}(t)}{dt}=PSc_{p}(t),$$

where *PS* is the permeability surface area, *F_p_* is the plasma flow and *c_a_* is the concentration in the supplying artery. The total concentration measured (*C*) is the combination of concentration in the plasma and the extracellular and extravascular volume:

(3) $$C\left(t\right)=v_{p}c_{p}\left(t\right)+v_{e}c_{e}(t).$$

The analytical solution to the CTU kinetic model has been shown previously to have the following form 9:

(4) $$C\left(t\right)=c_{a}(t)⊗(F_{p}e^{-t/T_{p}}+K^{trans}\left(1-e^{-t/T_{p}}\right)),$$

where ⊗ is the convolution operator, *T_p_* is the plasma transit time also given as the ratio $T_{p}=\frac{v_{p}}{F_{p}+PS}$, *E* is the extraction fraction also given as $E=\frac{PS}{PS+F_{p}}$, and *K^trans^* is the volume transfer constant and can be written as $K^{trans}=E\cdot F_{p}$.

### Linear Solution

By combining the coupled set of linear differential Equations (1) and (2) with Equation (3), we may derive the linear solution to the CTU model.

Substitute in Equations (1) and (2) into the derivate of Equation (3):

(5) $$\begin{array}{l} \frac{dC\left(t\right)}{dt}=-PSc_{p}\left(t\right)+F_{p}\left(c_{a}\left(t\right)-c_{p}\left(t\right)\right)+PSc_{p}\left(t\right)\\ =F_{p}\left(c_{a}\left(t\right)-c_{p}\left(t\right)\right). \end{array}$$

Differentiating once more and substituting in $\frac{dc_{p}(t)}{dt}$ isolated from Equation (1) yields:

(6) $$\frac{d^{2}C(t)}{dt^{2}}=F_{p}\frac{dc_{a}(t)}{dt}-\frac{F_{p}}{v_{p}}\left(-PSc_{p}\left(t\right)+F_{p}(c_{a}\left(t\right)-c_{p}\left(t\right))\right).$$

Further isolating $c_{p}(t)$ in Equation (5) and inserting into Equation (6), we have:

(7) $$\frac{d^{2}C(t)}{dt^{2}}=F_{p}\frac{dc_{a}(t)}{dt}+\frac{F_{p}PS}{v_{p}}c_{a}\left(t\right)-\frac{F_{p}+PS}{v_{p}}\frac{dC(t)}{dt}$$

Integrating Equation (7) twice over time gives an equation of the form:

(8) $$C=-\alpha \bar{C}+\beta \bar{c_{a}}+\gamma \bar{\bar{c_{a}}},$$

where $\bar{C}$ and $\bar{c_{a}}$ denote the integral of C and $c_{a}$, respectively, over time and $\bar{\bar{c_{a}}}$ is the double integral of $c_{a}$ over time. From the parameters ( α,β,γ) the following relations for ( $v_{p},\;F_{p},\;PS,T_{p},\;E$) may be found:

(9) $$v_{p}=\frac{\beta^{2}}{\alpha \beta -\gamma },\;F_{p}=\beta ,\;PS=\frac{\gamma \beta }{\alpha \beta -\gamma }\;,\;T_{p}=\frac{1}{\alpha },\;E=\frac{\gamma }{\alpha \beta }\;\text{.}$$

Equation (8) is a linear equation and may be expressed in matrix form as

(10) c=Ab.

The least‐squares solution can be found by solving the following problem using standard techniques:

(11) $$\underset{b}{\text{min}}||Ab-c|{|}^{2}$$

In the context of the CTU model, **A**, **b**, and **c** would be given as

(12) $$A=\left[\begin{array}{ccc} -\bar{C}(t_{0}) & \bar{c_{a}}(t_{0}) & \bar{\bar{c_{a}}}(t_{0})\\ -\bar{C}(t_{1}) & \bar{c_{a}}(t_{1}) & \bar{\bar{c_{a}}}(t_{1})\\ \vdots & \vdots & \vdots \\ -\bar{C}(t_{N-1}) & \bar{c_{a}}(t_{N-1}) & \bar{\bar{c_{a}}}(t_{N-1}) \end{array}\right]$$

$$b=\left[\begin{array}{c} \frac{PS+F_{p}}{v_{p}}\\ F_{p}\\ \frac{F_{p}PS}{v_{p}} \end{array}\right]\;i.e,\;\left[\begin{array}{c} \alpha \\ \beta \\ \gamma \end{array}\right]$$

and

$$c=\left[\begin{array}{c} \begin{array}{c} C(t_{0})\\ C(t_{1}) \end{array}\\ \begin{array}{c} \vdots \\ C(t_{N-1}) \end{array} \end{array}\right],$$

where *N* is the number of time points. Because *γ* is proportional to the extraction fraction *E*, which is defined for 0 ≤ *E* ≤ 1 a solution with *γ* ≈ 0 is consistent with a one‐compartmental state where either; no contrast agent extravasates, the contrast agent exchanges rapidly or the tissue is weakly vascularised 15.

## METHODS

### Simulation Data

**Figure 1:**
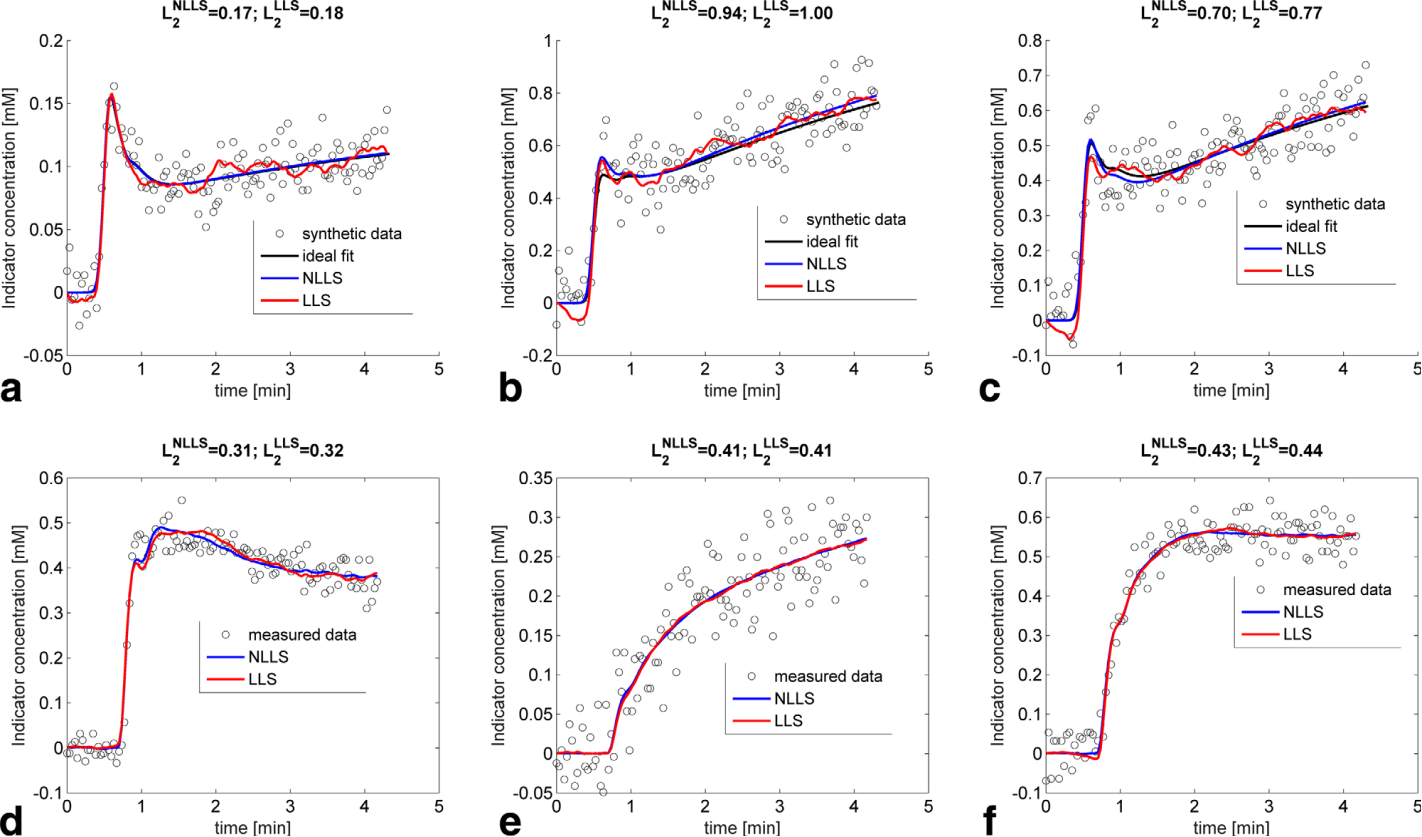
Example of the differences in fits between LLS and NLLS. (**a**–**c**) CNR was fixed at 10 and the temporal sampling at 2 s. The corresponding parameters were extracted from (a) Sourbron et al. 9, (b) Kallehauge et al. 23, and (c) Donaldson et al. 5 as summarised in Table 1. The L2‐norm showed slightly superior fits of NLLS over LLS for the simulated curves. (**d**–**e**) Clinical data curves reflecting different types of enhancement. The corresponding parameter estimates for NLLS and LLS were as follows: (d) *F_p_* (NLLS) = 0.76 min^−1^, *F_p_* (LLS) = 0.72 min^−1^, *v_p_* (NLLS) = 0.26 min^−1^, *v_p_* (LLS) = 0.26 min^−1^, *PS* (NLLS) = 0.03 min^−1^, *PS* (LLS) = 0.03 min^−1^. (e) *F_p_* (NLLS) = 0.11 min^−1^, *F_p_* (LLS) = 0.11 min^−1^, *v_p_* (NLLS) = 0.17 min^−1^, *v_p_* (LLS) = 0.18 min^−1^, *PS* (NLLS) = 0.06 min^−1^, *PS* (LLS) = 0.06 min^−1^. (f) *F_p_* (NLLS) = 0.48 min^−1^, *F_p_* (LLS) = 0.50 min^−1^, *v_p_* (NLLS) = 0.36 min^−1^, *v_p_* (LLS) = 0.35 min^−1^, *PS* (NLLS) = 0.05 min^−1^, *PS* (LLS) = 0.06 min^−1^. The L2‐norm shows very similar fit quality on the clinical data.

Synthetic concentration curves were generated (Fig. 1a–c) using Equation (4) with a noiseless input function from Parker et al. 16 with a 20‐s baseline and 4‐min postinjection duration. The temporal resolution was initially set at 10 ms before downsampling, and Gaussian noise was added to the tissue concentration curve C and $c_{a}$ to imitate more realistic clinical scenarios. The data were downsampled to a range of temporal resolutions in the interval Δt = (0.05–10 s) in 0.05‐s increments, and the onset time was varied randomly within the chosen resolution (Δt) for all simulations. The CNR was defined as the maximum difference in indicator concentration in the tissue divided by the standard deviation of the baseline noise and was simulated over a range of values (CNR values ranging from 2 to 40). The convolution operation was performed explicitly assuming an exponential decay for one of the functions as previously shown by Flouri et al. 12. Three different tissue enhancement curves were considered, corresponding to previously reported values extracted using the CTU model (summarised in Table 1).

**Table 1:** Parameters Used for Simulation.

	*F_p_* (min^−1^)	*v_p_*	*PS* (min^−1^)	Reference
Brain (tumor)	0.23	0.05	0.02	Sourbron et al. 9
Cervix (tumor)	0.57	0.28	0.2	Kallehauge et al. 23
Cervix (tumor)	0.65	0.22	0.14	Donaldson et al. 5

The simulations were performed using MATLAB (MathWorks, Natick, Massachusetts, USA) on an Intel Xeon 2‐core (2.4 GHz) with 20 GB RAM, and computation time was measured using the functions *tic()* and *toc()*. For the nonlinear parameter estimation, the *lsqnonlin()* function in MATLAB along with the Trust Region Reflective algorithm with bounds fixed for the *F_p_*, *PS*, and *v_p_* parameters to be real positive values 17 was used (and is referred to hereafter as NLLS). The initial starting guess supplied for NLLS was chosen to be the “true” values used to generate the synthetic data in order to avoid convergence to an unwanted local minimum. This means giving the NLLS a best case scenario with respect to parameter estimation and time to convergence, which may not reflect clinical reality. In other words, we compare the best possible performance of the NLLS with that of the linear least squares (LLS). The LLS solution was implemented by first calculating all the inputs for **A** (Equation (12)) using trapezoidal integration. The solution vector **b** was subsequently determined using analytical matrix inversion of the 3‐by‐3 matrix **A^T^A**, where **A^T^** is the transpose of **A**. The goodness‐of‐fit was compared using the Euclidean distance (L2‐norm) between the data and the fit as estimated from Equations (4) and (8) for NLLS and LLS, respectively. All simulation code can be found online at https://github.com/Jkallehauge/Linear‐CTU.

### Statistical Analysis

A Monte Carlo simulation of 1000 runs with different random noise was performed for each condition of temporal downsampling and noise level. For each of the 1000 runs, the estimated values for *F_p_*, *PS*, and *v_p_* were calculated using the linear and nonlinear approach and compared. The systematic and stochastic variation in parameter estimations were evaluated using precision and percentage error, which is here defined as

$$Precision\left(\%\right)=\frac{\sigma }{\mu }\times 100$$

$$Error\left(\%\right)=\frac{\bar{x-\mu }}{\mu }\times 100,$$

where *x* is the parameter value for each of the 1000 simulations, *σ* is the standard deviation of *x*, and *μ* is the “true” value from which the synthetic data have been generated.

### Clinical Data

The clinical applicability was investigated in a prospective study approved by the local medical ethics research board, with written informed consent from all patients. A total of 14 patients with locally advanced cervical cancer were scanned within one week of the start of chemoradiotherapy using MRI on a 3T Philips Achieva‐X scanner. DCE‐MRI was performed using a three‐dimensional axial nonselective saturation recovery spoiled gradient echo technique with the following parameters: number of slices = 20; slice thickness = 5 mm; repetition time = 2.9 ms; echo time = 1.4 ms; *T_sat_* = 25 ms; flip angle = 10°; in‐plane resolution = 2.3 × 2.3 mm; and time resolution = 2.1 s. The bolus injected was 0.1 mmol/kg Dotarem at 4 mL/s, followed by a 50‐mL saline flush. A total of 120 dynamic scans were obtained, of which an average of 18 time points were scanned before the bolus arrived at the external iliac arteries. A T1 relaxation map was constructed [following Deoni et al. 18] before contrast agent injection using a three‐dimensional gradient recalled echo sequence with five different flip angle scans (5°, 10°, 15°, 20°, 25°) with the same orientation and field of view as the dynamic scan, a repetition time of 20 ms, and an echo time of 1.7 ms. The dynamic magnitude images were subsequently converted into contrast agent concentrations as described previously 19. The regions of interest were chosen to be the clinical gross tumor volume delineated by an experienced oncologist on a transversal T2‐weighted MRI, following the recommendations of the GEC‐ESTRO working group 20. In each patient, an arterial input function (AIF) was derived by averaging the measured voxelwise AIFs over a number of included voxels in the left femoral arteries where the B1 field was consistently most homogenous (inspected qualitatively). Specifically for the AIF, the precontrast longitudinal relaxation (T_1,0_) determination was connected with some uncertainty, and a literature value for T_1,0_ was chosen instead: T_1,0_(blood) = 1660 ms 21. For the remaining tissue curves, the estimated T1‐map was used for the conversion from signal to contrast concentration. To correct for differences in large and small vessel hematocrit, the AIF was multiplied by a factor 1.18, based on an assumed hematocrit of 0.38 and an assumed small‐to‐large vessel ratio of 0.7 22. The clinical data were analysed using both LLS and NLLS.

## RESULTS

### Synthetic Data

Figure 1a–c shows three example simulations using a temporal resolution of 2 s, where the total acquisition time was 260 s with a baseline of 20 s, CNR = 10, and kinetic parameters corresponding to those in Table 1. Both the NLLS and LLS fits described the synthetic data similarly with only a small difference in the L2‐norm ( $L_{2}^{NLLS}$ and $L_{2}^{LLS}$).

**Figure 2:**
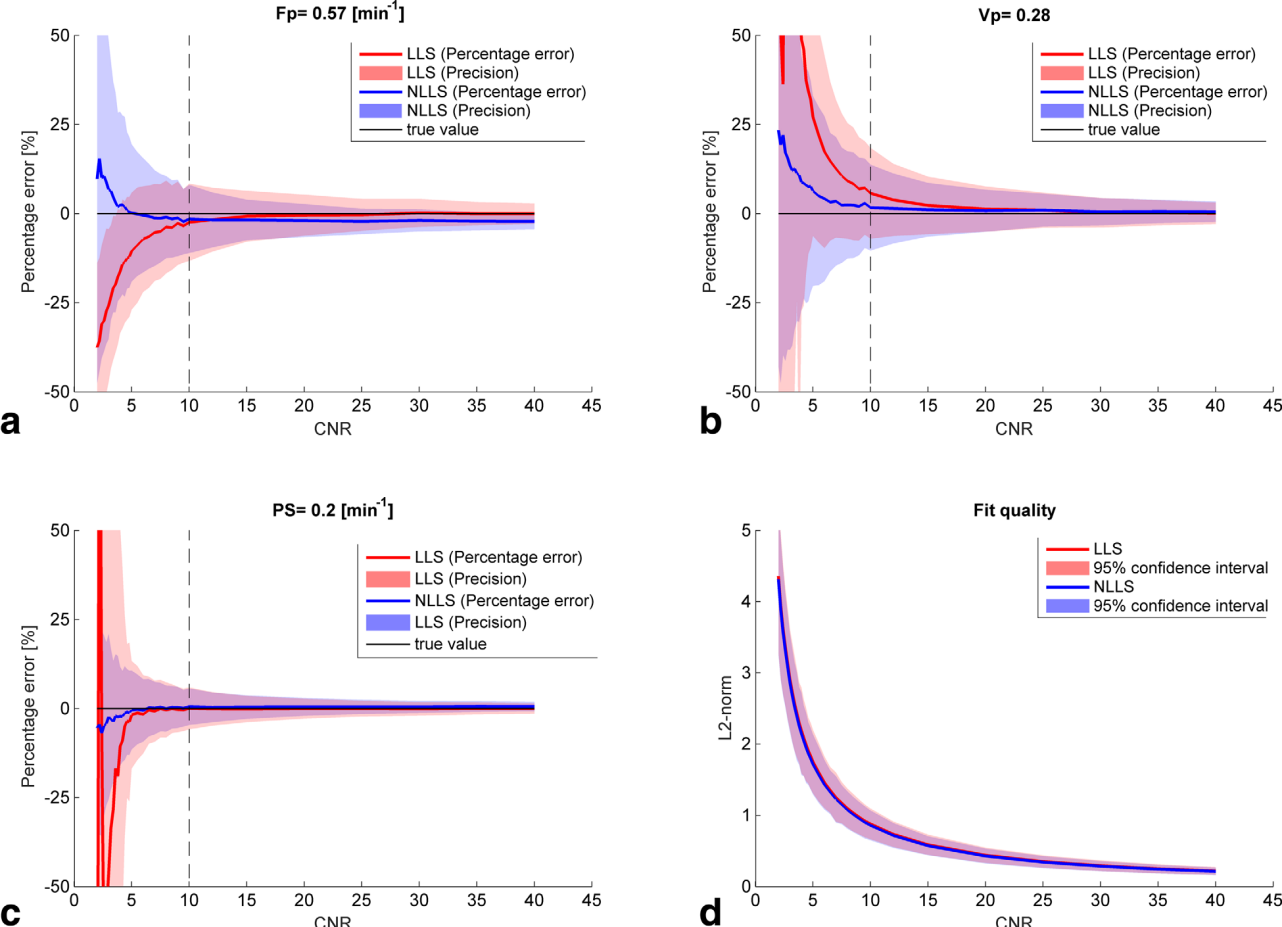
Influence of noise on the percentage error and precision of each hemodynamic parameter (**a**–**c**) and the overall fit (**d**) when applying both NLLS and LLS. The vertical black lines correspond to the values shown in Table 2 (middle row).

The difference between the true values and derived parameters using both NLLS and LLS under varying noise conditions are summarised in Figure 2. Figure 2a shows the results for *F_p_* where the LLS underestimates the true value of *F_p_* under noisy conditions (CNR < 10), whereas NLLS tends to overestimate *F_p_* under very noisy conditions (CNR < 5). At high CNR values, LLS approximates the true value better than NLLS. The precision of estimating *F_p_* was consistently better for NLLS for CNR > 10. Both NLLS and LLS overestimated the true value of *v_p_* (Fig. 2b), although NLLS less so than LLS for low CNR (CNR < 15). The two curves converge around CNR ≈ 15 after which little difference between the two curves is seen both in terms of percentage error and precision. The influence of noise on PS was also lower for NLLS compared with LLS under low noise conditions, and above CNR = 10 their precision and percentage error were comparable (Fig. 2c). The overall effect of noise on the quality of the fit (Fig. 2d) was measured using the L2‐norm and showed comparable performance across all CNR values.

The percentage error and precision for the three different tissue types (see Table 1) are summarised in Table 2 for a realistic temporal resolution and noise level (Δt = 2 s and CNR = 10, corresponding to the vertical dashed lines in Fig. 2). Both NLLS and LLS underestimated the true values of *F_p_* for the three tissue types, with only marginally better precision and percentage error for NLLS over for LLS. *v_p_* was generally overestimated across all simulated tissues, however, to a lesser degree for NLLS. The precision of $v_{P}$ was again better for NLLS. The percentage error and precision of *PS* estimated using both NLLS and LLS were comparable and with almost no bias.

**Table 2:** Percentage Error and Precision for Different Tissue Types at Δt = 2 s and CNR = 10.

	*F_p_* (min^−1^)		*v_p_*		*PS* (min^−1^)	
	NLLS	LLS	NLLS	LLS	NLLS	LLS
Brain (tumor)	−0.2 (7.4)	−3.7 (10.0)	−0.1 (4.8)	2.0 (5.3)	−0.3 (4.6)	−0.7 (5.0)
Cervix (tumor)	−1.6 (9.2)	−1.9 (10.6)	1.7 (12.0)	5.5 (12.7)	0.3 (5.0)	−0.5 (5.7)
Cervix (tumor)	−1.7 (8.1)	−2.7 (9.7)	0.6 (7.5)	3.4 (8.0)	0.1 (3.9)	−0.3 (4.5)

**Figure 3:**
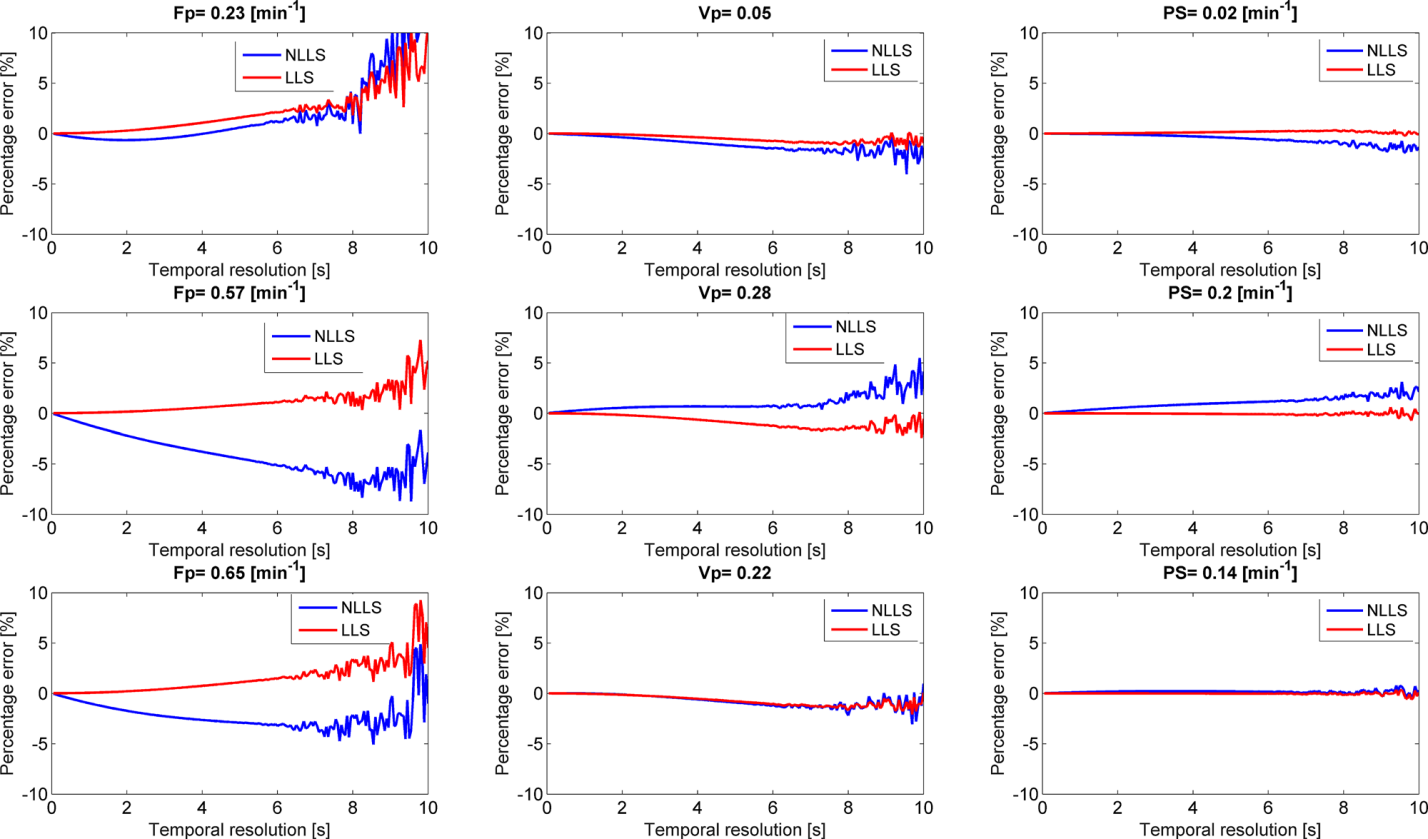
Effect of temporal downsampling on both LLS and NLLS for the three different simulated tissue types from Table 1.

The effect of temporal downsampling as examined on noiseless data is shown in Figure 3. For the higher tested tissue values of *F_p_* (0.57 min^−1^ and 0.65 min^−1^), LLS was less influenced by temporal downsampling than NLLS while for the low value $F_{p}$ (0.23 min^−1^) NLLS was less influenced. Almost no influence of temporal sampling was observed for $v_{p}$ and PS for lower temporal sampling. Above a temporal period of 8 s, both LLS and NLLS begin to show oscillations corresponding to the width of the Parker input function (≈8.4 s) 16. The speed improvement of the LLS approach was, over all temporal resolutions and tested parameter configurations, at minimum around 230 times faster than the NLLS (Supporting Fig. S1).

### Clinical Data

**Figure 4:**
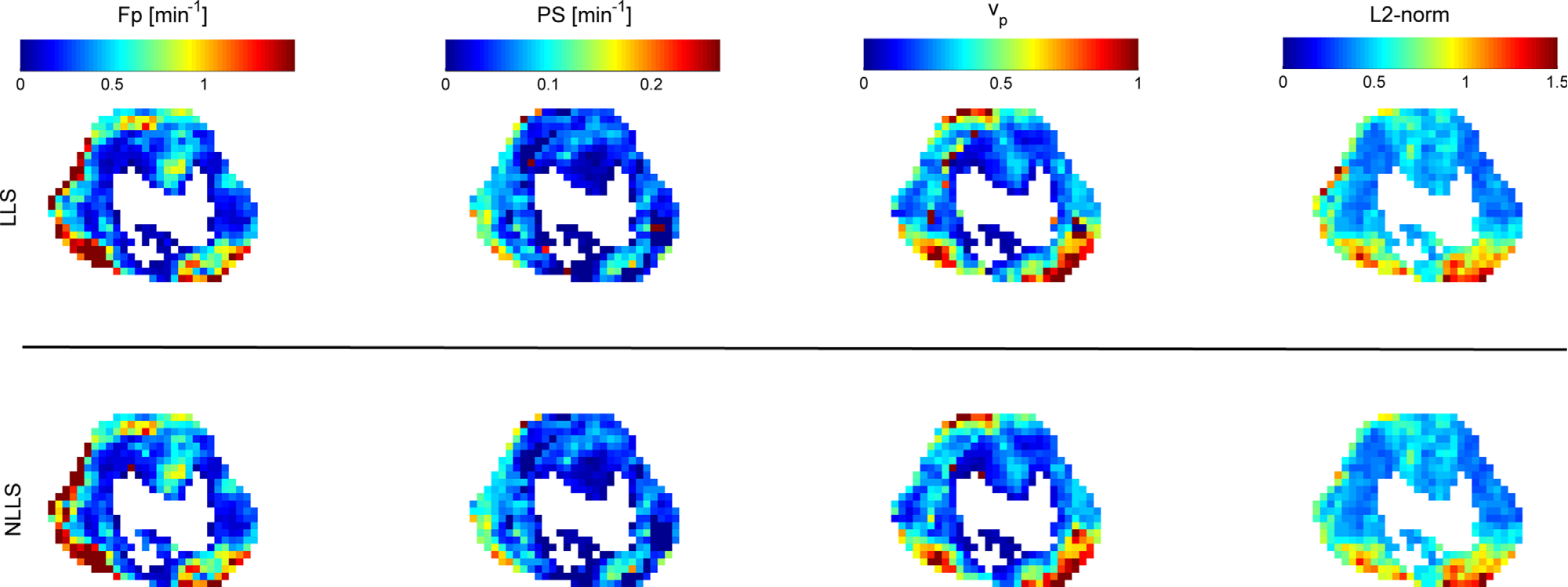
Comparison of hemodynamic maps estimated and goodness‐of‐fit using both NLLS and LLS. The white center corresponds to data with negative distribution volume or a fit completely contained within the 95% confidence interval of the baseline noise.

Figure 1d–f shows three clinical example curves with little difference in the performance of the LLS and NLLS. The three curves were chosen to reflect different capillary transit times: fast (Fig. 1d), slow (Fig. 1e), and intermediate (Fig. 1f). Figure 4 shows a comparison of the kinetic maps derived using both LLS and NLLS for a single central slice through a patient's tumor. The maps show very similar patterns, suggesting the two approaches can be used interchangeably (see also Supporting Figure S3a–n).

**Figure 5:**
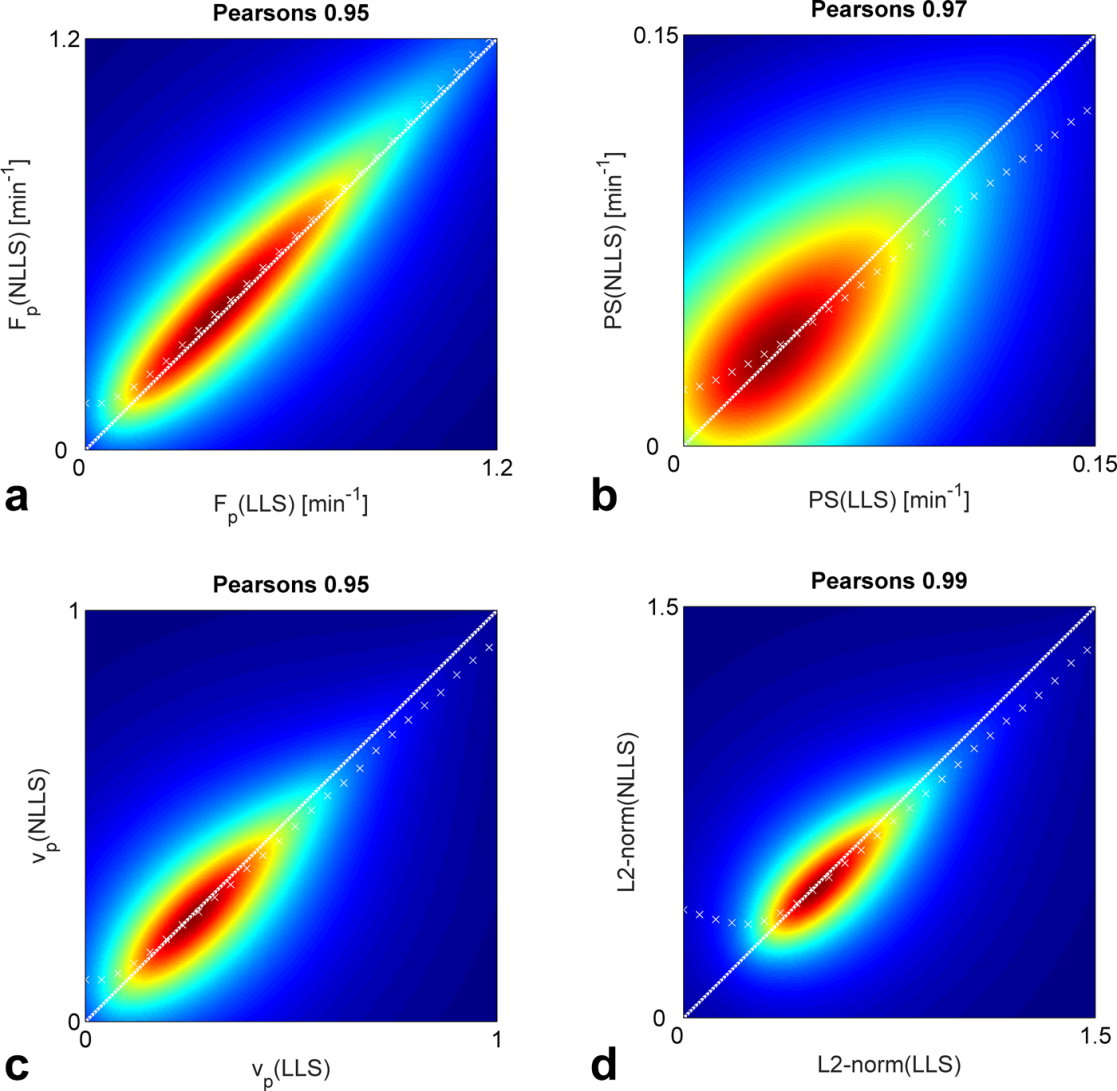
Correlations between LLS and NLLS parameters and fit residuals for all voxels where *v_p_* > 0 and 0 ≤ *E* ≤ 1. The white dashed lines are the identity line and the white cross marks (×) show the mode (most frequent) corresponding parameter estimates.

**Figure 6:**
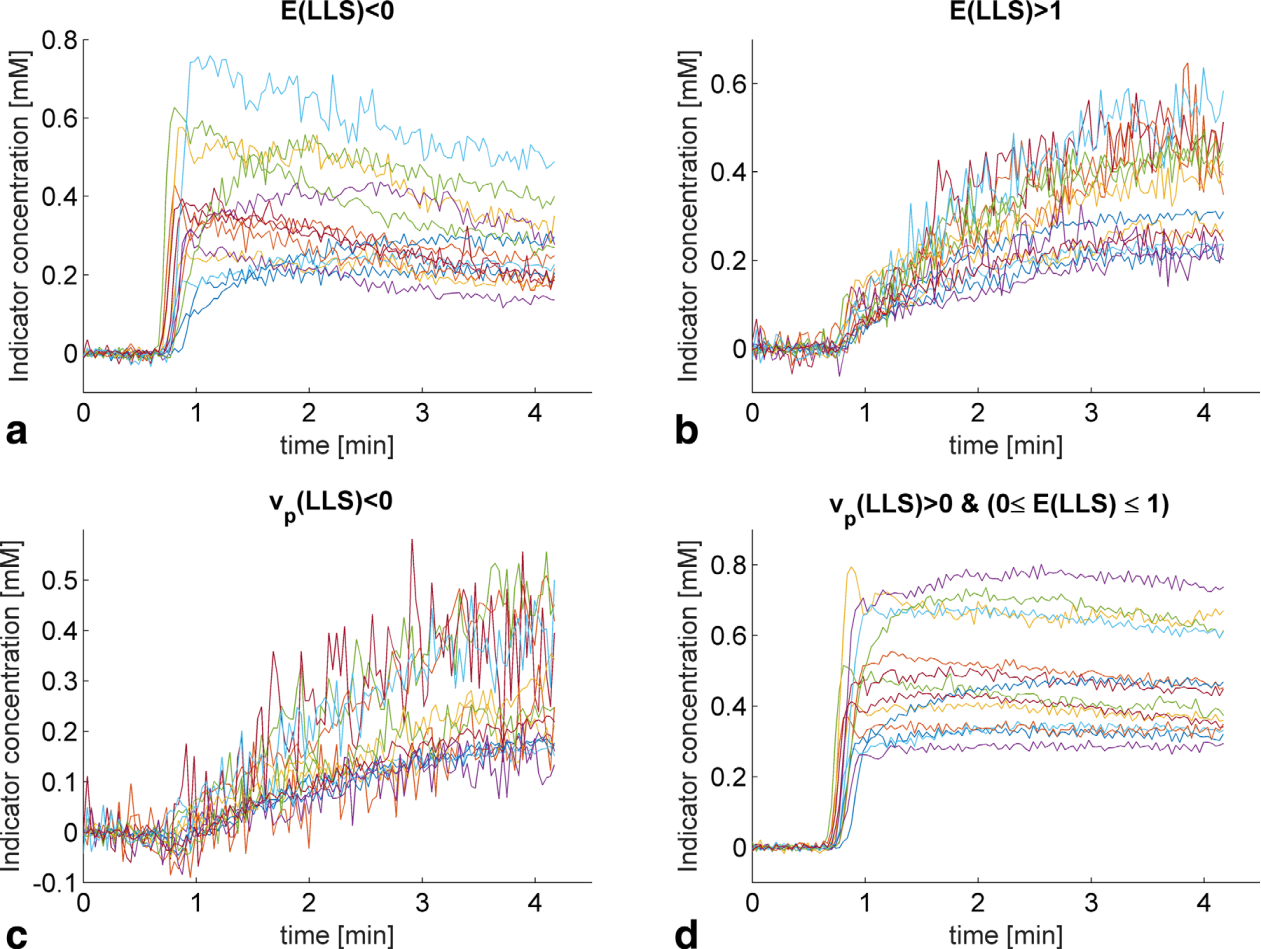
Patient‐wise median uptake curves for the different regions within the tumor tissue. (**a**–**c**) Curves that were excluded from the final comparison between NLLS and LLS. (**d**) Curves that were compared. (**a**) Curves that had a negative extraction fraction appear to have significant washout. (**b**) Curves that had an extraction fraction greater than one appear to be enhancing slowly. (**c**) Curves that had a negative plasma volume fraction appear to be enhancing slowly with a slight decrease in concentration initially. (**d**) Curves that had a positive plasma volume fraction and an extraction fraction between 0 and 1. The noise on these curves is less due the greater number of curves used for calculating the median curves.

By aggregating the data from all 14 patients, a total of 34,525 tumor voxels were analysed using both LLS and NLLS (see overview in Supporting Fig. S2). A subset of these voxels were excluded if they had a negative distribution volume ( $v_{d}=\int^{}C(t)dt/\int^{}c_{a}(t)dt$) or the model fit was completely contained within the 95% confidence interval of the baseline noise. The remaining 32,190 voxels had a median CNR of 17.4 (95% confidence interval: 6.9, 35.8). LLS returned unrealistic negative values of *F_p_*, *PS*, and *v_p_* in a considerable proportion of the 32,190 voxels, whereas the NLLS was constrained to be within the set boundary points of the fitting algorithm. Discarding the regions where the CTU model is not defined (i.e., where *v_p_* < 0 or *E* was not between zero and 1 as estimated by LLS [25,325 voxels left]), we found good agreement between NLLS and LLS (Fig. 5). The white dashed line shows the identity line, and the white cross marks (×) illustrate where the mode or most frequent parameters are seen, thus indicating whether LLS over‐ or underestimates compared with NLLS within in a given range of parameters. Figure 5a generally shows that LLS and NLLS agreed well over a large range of *F_p_*, and only at low values does it suggest that *F_p_* is overestimated by NLLS compared with LLS. Similar findings are seen in Figure 5b,c for *PS* and *v_p_*, although they also suggest that LLS overestimates the parameter values at high values compared with NLLS. The L2‐norm also showed good agreement with a Pearson's correlation of 0.99. The excluded voxels from the final comparison between the NLLS and LLS were investigated further. Supporting Figure S4 shows example curves of the regions with negative extraction fraction, regions with extraction fraction greater than one, and regions with negative plasma volume fraction. Similarly, Figure 6 shows the patient‐wise median curves of the aforementioned regions along with the patient‐wise median curves of the data included in the comparison of NLLS and LLS. Generally, the regions with *E* (LLS) < 0 showed significant washout, whereas the regions with *E* (LLS) > 1 showed slow enhancement, which may be described adequately by a more simple model (e.g., a one‐compartment model). The regions that had a negative plasma volume fraction again showed slow enhancement, with a slight decrease in the initial indicator concentration. A quantitative comparison of the different parameters in the different regions can be found in Table 3. Here, the CNR level was seen to be lower in the regions excluded compared with the included regions. The plasma transit time estimated by NLLS [T_p_ (NLLS)] was furthermore considerably longer in the regions where *E* (LLS) > 1 or *v_p_* (LLS) < 0. Whereas *E* (LLS) < 0, the *T_p_* (NLLS) was comparable to that of the included voxels, although with a much larger confidence interval. A similar result was observed for *F_p_* (NLLS). Where *v_p_* (LLS) was negative, *v_p_* (NLLS) similarly returned unrealistic values with a median that was greater than 1.

**Table 3:** Characteristics of Included and Excluded Voxels from Clinical Data.

	*E* (LLS) < 0	*E* (LLS) > 1	*v_p_* (LLS) < 0	*v_p_* (LLS) > 0 ∩ (0 ≤ E (LLS) ≤ 1)
*F_p_* (NLLS) (min^−1^)	0.42 (0.03, 3602)	0.10 (0.02, 0.71)	0.05 (0.01, 0.21)	0.56 (0.11, 2.14)
*F_p_* (LLS) (min^−1^)	0.29 (−0.38, 1.97)	0.07 (0.00, 0.32)	0.03 (−0.12, 0.20)	0.54 (0.09, 1.95)
*PS* (NLLS) (min^−1^)	2.2e‐12 (2.2e‐14, 0.08)	3.3e‐11 (2.2e‐14, 5.1)	0.0 (2.60e‐14, 225)	0.05 (0.00, 0.19)
*PS* (LLS) (min^−1^)	−0.01 (−0.14, 0.04)	−0.28 (−8.80, −0.00)	0.05 (−0.18, 10.29)	0.04 (0.00, 0.18)
*v_p_* (NLLS)	0.24 (0.02, 63.7)	0.42 (0.01, 100)	45.7 (0.1, 100)	0.30 (0.06, 0.86)
*v_p_* (LLS)	0.25 (−0.01, 1.18)	0.68 (−0.34, 4.18)	−0.13 (−15.02, −0.00)	0.31 (0.07, 0.90)
*E* (NLLS)	5.7e‐12 (1.4e‐14, 0.2)	3.4e‐10 (1.1e‐13, 0.98)	0.01 (3.4e‐13, 0.99)	0.08 (0.01, 0.31)
*E* (LLS)	−0.05 (−7.27, −0.00)	1.41 (1.01, 30.5)	0.84 (−19.7, 18.04)	0.08 (0.01, 0.32)
*K^trans^* (NLLS)	2.2e‐12 (2.2e‐14, 0.07)	3.3e‐11 (2.2e‐14, 0.10)	0.00 (2.6e‐14, 0.04)	0.04 (0.00, 0.15)
*K^trans^* (LLS)	−0.01 (−0.53, 0.08)	0.11 (0.01, 2.2)	0.06 (−0.14, 0.34)	0.04 (0.00, 0.15)
*T_p_* (NLLS)	0.46 (8.e‐06, 1295.0)	3.49 (0.03, 3185.67)	860.6 (0.1, 5268.7)	0.47 (0.15, 1.34)
*T_p_* (LLS)	0.63 (−0.33, 17.08)	−2.2 (−79.6, 6.8)	−0.55 (−20.00, 12.16)	0.52 (0.19, 1.55)
CNR	12.0 (4.5, 30.1)	10.2 (4.4, 22.8)	8.5 (4.3, 18.9)	17.4 (6.9, 35.8)

All data are presented as the median (95% confidence interval).

## DISCUSSION

### Principal Findings

In this study, we derived and evaluated the precision and percentage error of a linearised CTU kinetic model. Specifically, we compared the effects of temporal downsampling, varying noise, and different tissue hemodynamic parameters on the precision and percentage error of both a nonlinear and linear CTU model. Within the clinical achievable ranges (CNR ≈ 10) and temporal resolution (Δt ≈ 2 s) 23, LLS showed comparable performance in terms of percentage error and precision compared with NLLS. Parameters estimated using LLS were generally more stable to temporal downsampling (Fig. 3), whereas NLLS was consistently more stable to variations in noise (Fig. 2). The clinical comparison of LLS and NLLS showed very high agreement in parameter estimation (Fig. 5). The simulations and the clinical data analysis agreed in that LLS and NLLS performed comparably under sufficiently high CNR and at the sampling rate for the clinical data (2.1 s).

### Interpretation of Findings

Previous studies have focused on the effects of temporal downsampling on the accuracy of hemodynamic parameters extracted from the Tofts model 24 and the 2CXM 7. These studies showed that the amplitudes of the impulse response functions of the Tofts model and 2CXM (i.e., *K^trans^* and *F_p_*) were underestimated with increasing temporal downsampling, whereas the distribution volumes (i.e., *v_e_* and *v_e_* + *v_p_*, respectively) were overestimated. We found a similar trend for *F_p_* for the cervical cancer simulation data; however, we found very little effect of temporal downsampling on the distribution volume. The effect of temporal downsampling on *PS* was also investigated in the study of the 2CXM 7 where a slight overestimation was observed (for *PS* = 0.10 min^−1^), and again we found little evidence for this dependency. Our findings are more in line with Flouri et al. 12 for NLLS, possibly because of similar implementation of the convolution operation. Finally, if the temporal resolution Δt is comparable or larger than the peak width of *c_a_*, the quick wash‐in process in *c_a_* and *C* may be missed if the two sampling points happen to be at two distal ends of the first‐pass peak resulting in inaccurate calculation of the hemodynamic parameters. For the Parker input function, the full‐width half‐maximum is 8.4 s corresponding to the sudden changes in percentage error at the temporal sampling of around 8 s (Fig. 3). The improved computational efficiency for the linear CTU model has also been shown in the linear versions of the Tofts and extended Tofts models 11, 25 and 2CXM 12.

### Implications

One major weakness of nonlinear fitting algorithms is the problem of supplying a sensible initial guess in order for the algorithm to converge to the global minimum. It has been shown that simply using one set of initial guess parameters at the center of the parameter spaces is insufficient and will result in considerable errors in the fits, and therefore multiple start points are recommended 26. Because the linear CTU model identifies the global minimum directly, a concatenated scheme where the linear CTU model initializes the guess for the nonlinear CTU model may improve speed, accuracy, and precision. In our simulations, we deliberately chose the initial guess for NLLS to be the true values to avoid convergence to a local minima. In practice, the true values are never known and it would require multiple initialization for robust estimation of the pharmacokinetic parameters. This in turn means that the speed improvement of LLS over the NLLS noted here would in practice, be higher.

With the current level of MR scanner technology, CNR and temporal resolution, the linear CTU model may by itself be the most suitable method of obtaining hemodynamic parameters. However, in low enhancing tissue regions (with low CNR), the linear CTU model should be complemented by the nonlinear CTU model to obtain sufficient accuracy and precision.

### Limitations

A well‐known limitation of any linear formulation of a nonlinear problem is the incorrect accounting of experimental noise contributions. Where the nonlinear formulation (Eq. (4)) may correctly assume a normally distributed noise profile, once formulated in linear form (Eq. (8)), this may no longer be valid. Similar observations have been addressed in the linear calculation of T1 relaxation times, which results in a general overestimation of T1 values compared with the nonlinear formulation. Numerous ways of improving upon this bias have been proposed by multiplying appropriate weights to both sides of Equation (8) 27, 28 and have recently been successfully implemented for the 2CXM 12, resulting in improved accuracy and precision. However, the authors note that choosing the optimal weighting scheme is a nontrivial task and deserves a more in‐depth study. Further investigation in this direction may potentially improve the precision of the hemodynamic parameters estimated by the linear CTU model. Finally, the return of unrealistic values of the extraction fraction and the plasma volume fraction from the LLS resulted in exclusion of a considerable number of voxels from the final comparison of NLLS and LLS. In these regions, it is possible that the constrained NLLS would be able to extract more plausible kinetic parameter estimates, especially under noisy conditions. Conversely, the constrained NLLS could also mask problems, such artifacts in the data and unsuitable model choice, and create a false sense of confidence in the results.

## CONCLUSION

In this study, we have derived the linear version of the CTU kinetic model and compared its performance with the nonlinear CTU model, with varying noise and temporal downsampling. The linear CTU model has precision and percentage error comparable to the nonlinear within clinical achievable ranges of CNR and temporal resolution. The linear CTU model is computationally more efficient and more stable to temporal downsampling, whereas the nonlinear model is more robust to variations of noise.

## Supporting information


**Fig. S1**. Improved speed of LLS over NLLS as a function off the temporal resolution for three different combinations of F_p_, PS and v_p_.
**Fig. S2**. Overview of the voxel exclusion process. The initial pool of candidate voxels were excluded if the distribution volume (v_d_) was negative or if the CNR was within 95% of the baseline noise. Of the remaining voxels, a further subset was excluded if they had negative *v_p_* (LLS) or if *E* (LLS) was not inside the interval 0 and 1.
**Fig. S3a–S3n**. Center slice through tumor comparing estimated hemodynamic maps and goodness‐of‐fit using both NLLS and LLS (Patient 1–14).
**Fig. S4**. Example curves excluded from the comparison of NLLS and LLS. (**a**) Typical data excluded when *E* (LLS) < 0. (**b**) Typical data excluded when *E* (LLS) > 1. (**c**) Typical data excluded when *v_p_* (LLS) < 0. For comparison, we also included the fit of the one‐compartment model ( $C\left(t\right)=c_{a}(t)⊗(F_{p}e^{-t\cdot F_{p}/v_{p}}$)).
