# Supplementary material for: Comparison of linear and nonlinear implementation of the compartmental tissue uptake model for dynamic contrast‐enhanced MRI
Source: Magn Reson Med. 2016 Sep 8;77(6):2414–23. doi: 10.1002/mrm.26324 (PMC5484345; doi:10.1002/mrm.26324)
Supplement: Supplementary file 1 — Fig. S1. Improved speed of LLS over NLLS as a function off the temporal resolution for three different combinations of Fp, PS and vp. Fig. S2. Overview of the voxel exclusion process. The initial pool of candidate voxels were excluded if the distribution volume (vd) was negative or if the CNR was within 95% of the baseline noise. Of the remaining voxels, a further subset was excluded if they had negative vp (LLS) or if E (LLS) was not inside the interval 0 and 1. Fig. S3a–S3n. Center slice through tumor comparing estimated hemodynamic maps and goodness‐of‐fit using both NLLS and LLS (Patient 1–14). Fig. S4. Example curves excluded from the comparison of NLLS and LLS. (a) Typical data excluded when E (LLS) < 0. (b) Typical data excluded when E (LLS) > 1. (c) Typical data excluded when vp (LLS) < 0. For comparison, we also included the fit of the one‐compartment model ( C(t)=ca(t)⊗(Fpe−t⋅Fp/vp)). [file MRM-77-2414-s001.docx]

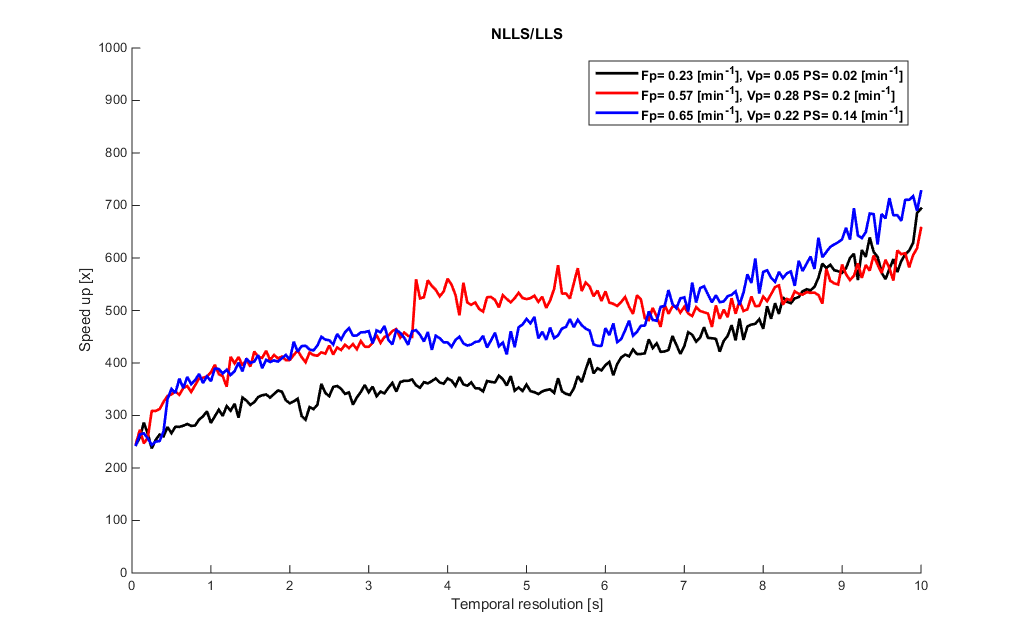


**Supporting Figure S1:** Improved speed of LLS over NLLS as a function off the temporal resolution for three different combination of F_p_, PS and v_p_.


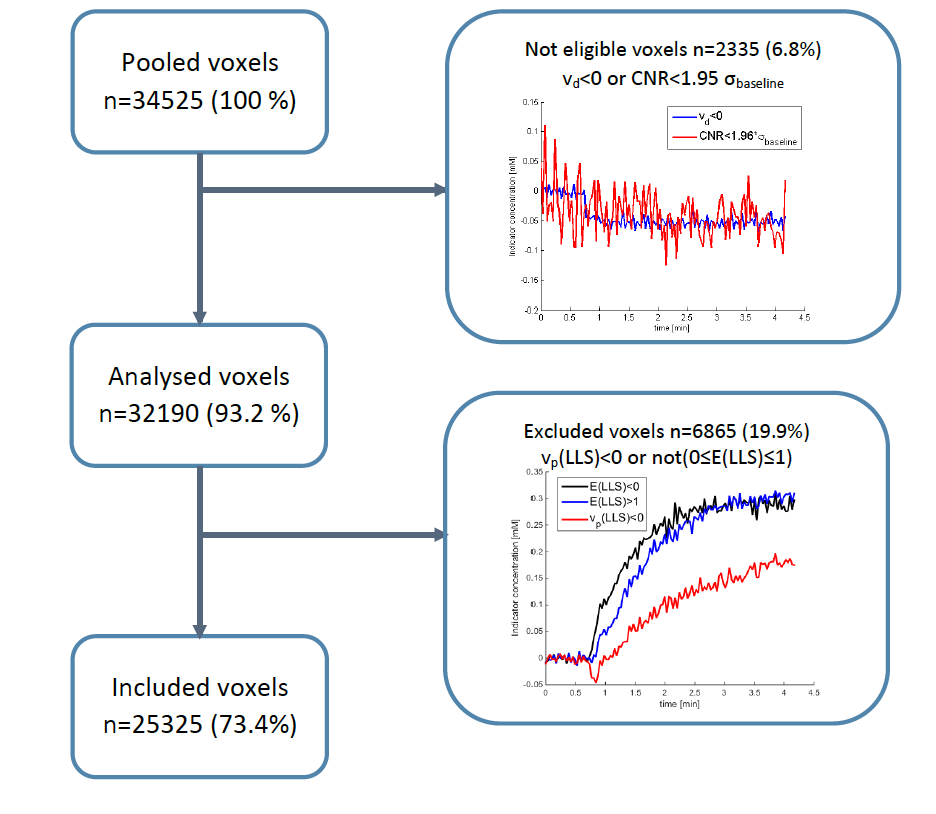


**Supporting Figure S2:** Overview of the voxel exclusion process. The initial pool of candidate voxels were excluded if the distribution volume (v_d_ was negative or the CNR was within 95% of the baseline noise). Of the remaining voxels a further subset was excluded of they had negative v_p_(LLS) or E(LLS) was inside the interval 0 and 1.


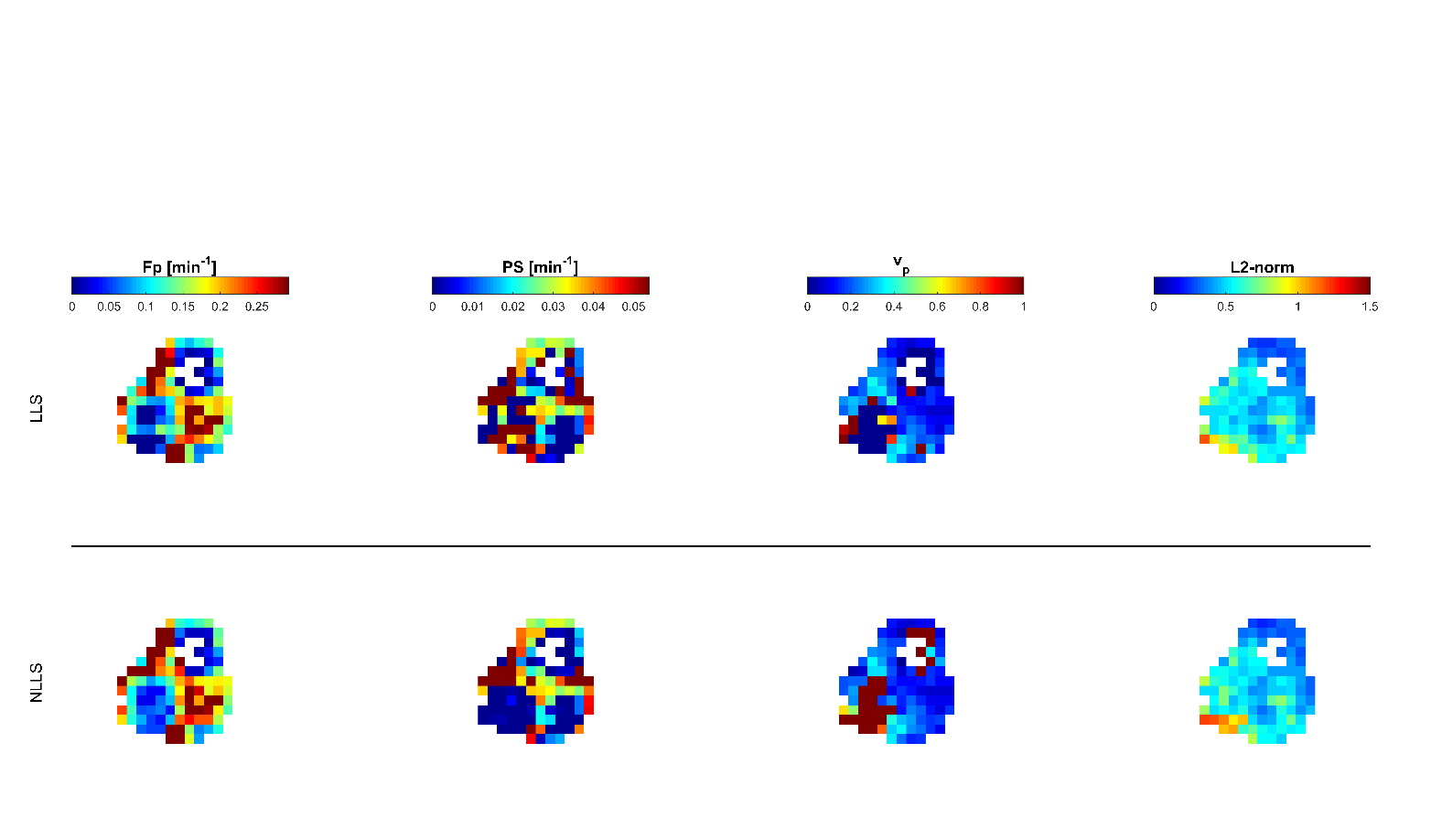


**Supporting Figure S3a:** Centre slice through tumour comparing estimated haemodynamic maps and goodness-of-fit using both NLLS and LLS (Patient 1).


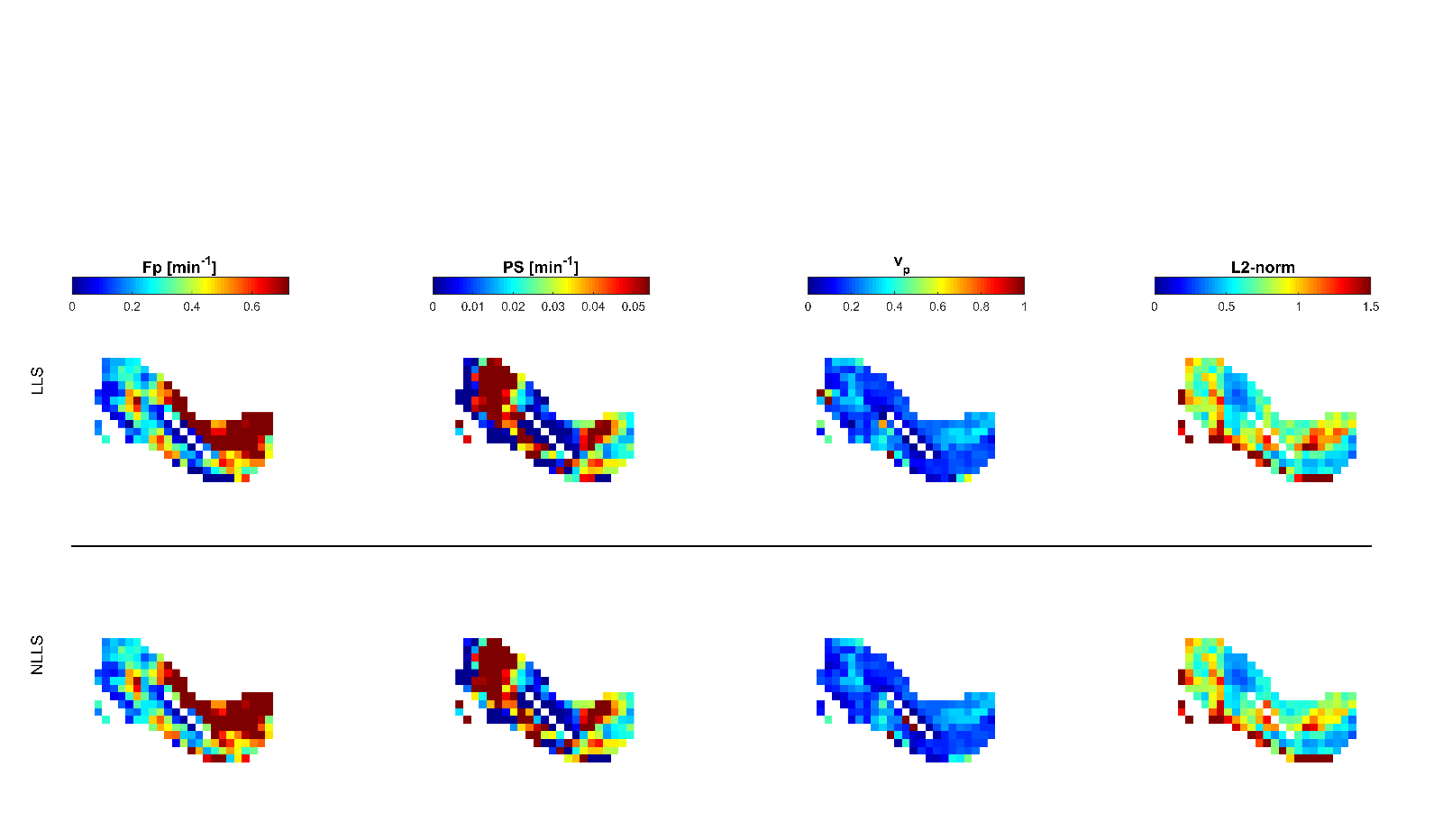


**Supporting Figure S3b:** Centre slice through tumour comparing estimated haemodynamic maps and goodness-of-fit using both NLLS and LLS (Patient 2).


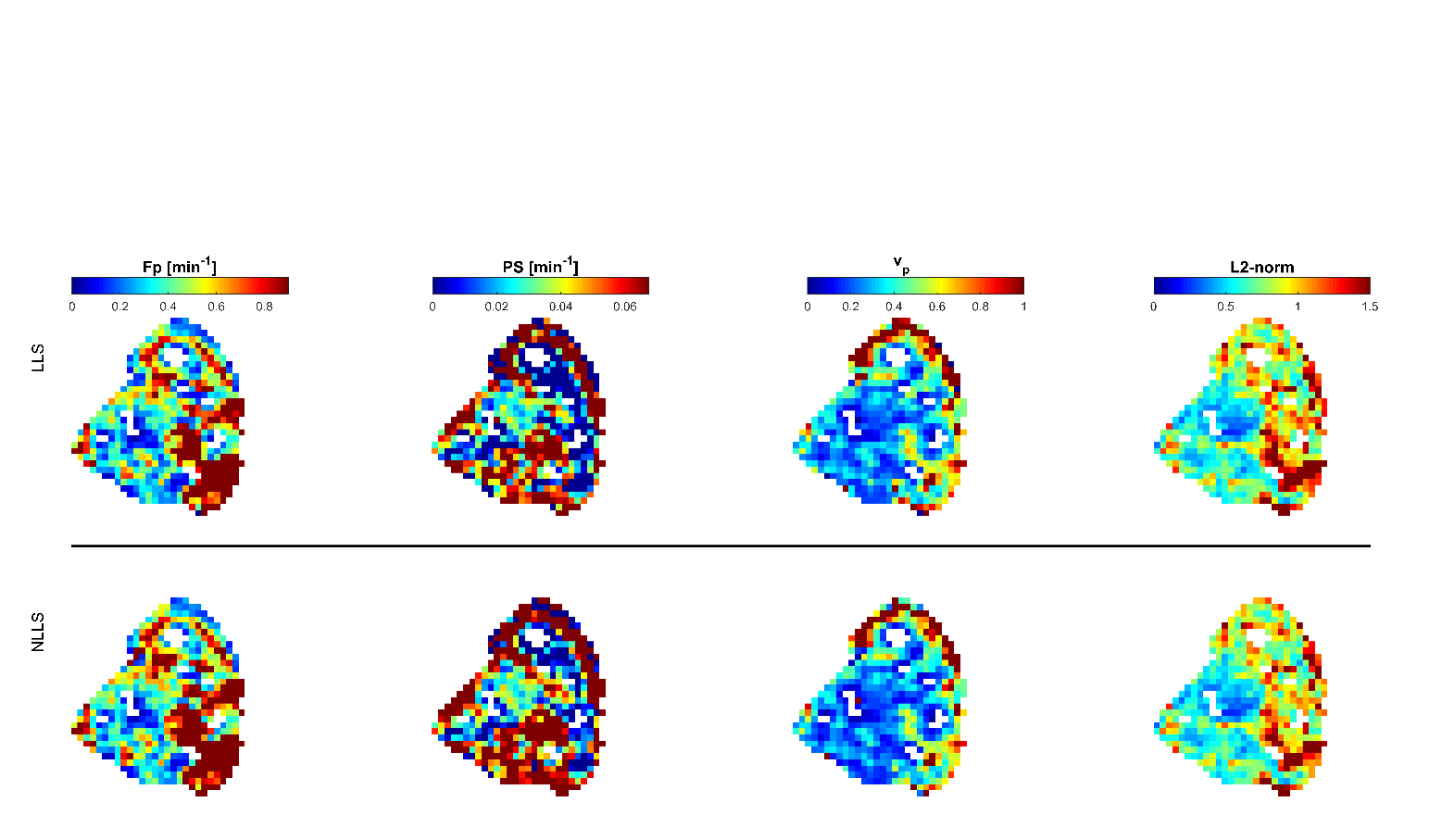


**Supporting Figure S3c:** Centre slice through tumour comparing estimated haemodynamic maps and goodness-of-fit using both NLLS and LLS (Patient 3).


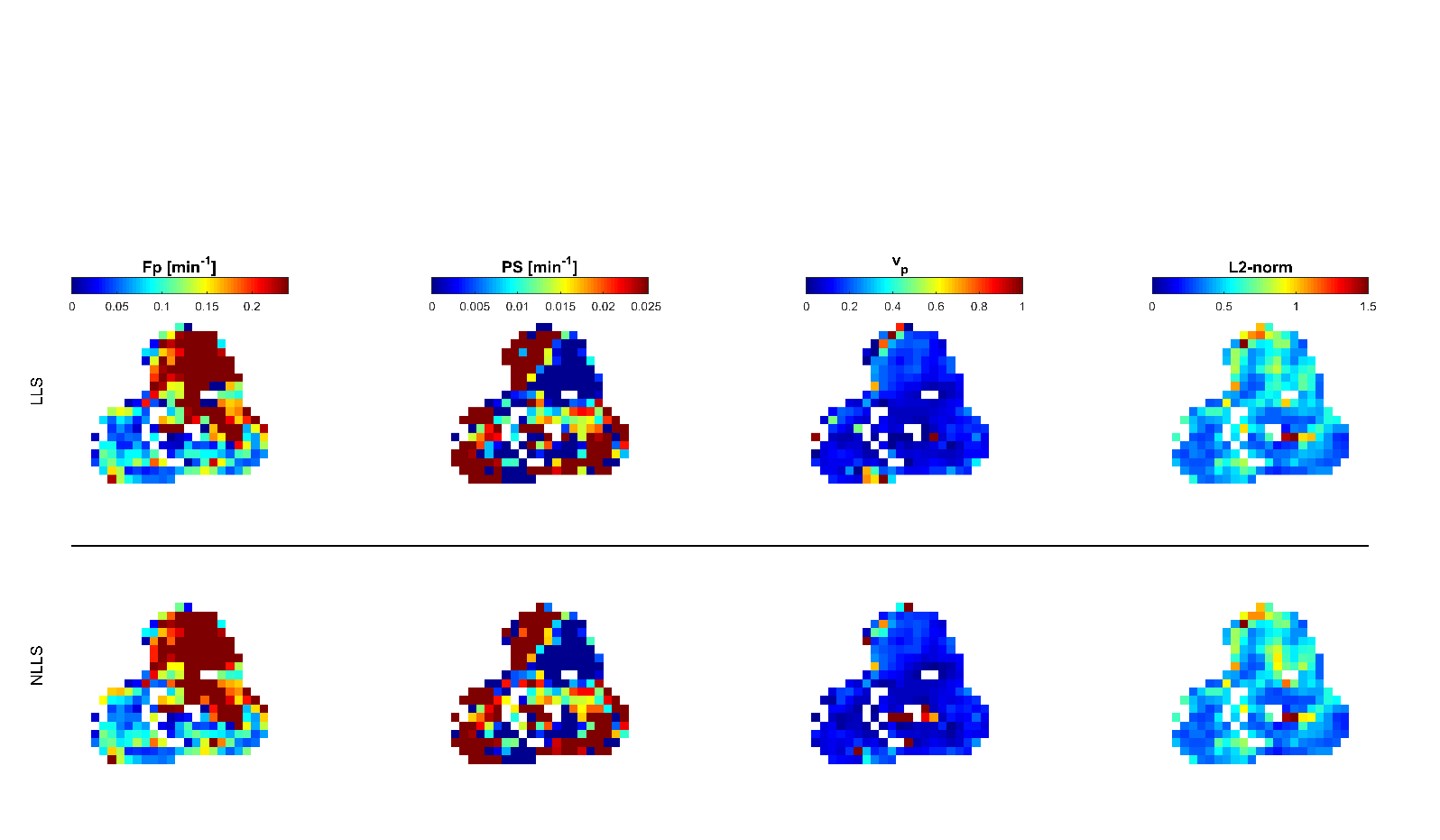


**Supporting Figure S3d:** Centre slice through tumour comparing estimated haemodynamic maps and goodness-of-fit using both NLLS and LLS (Patient 4).


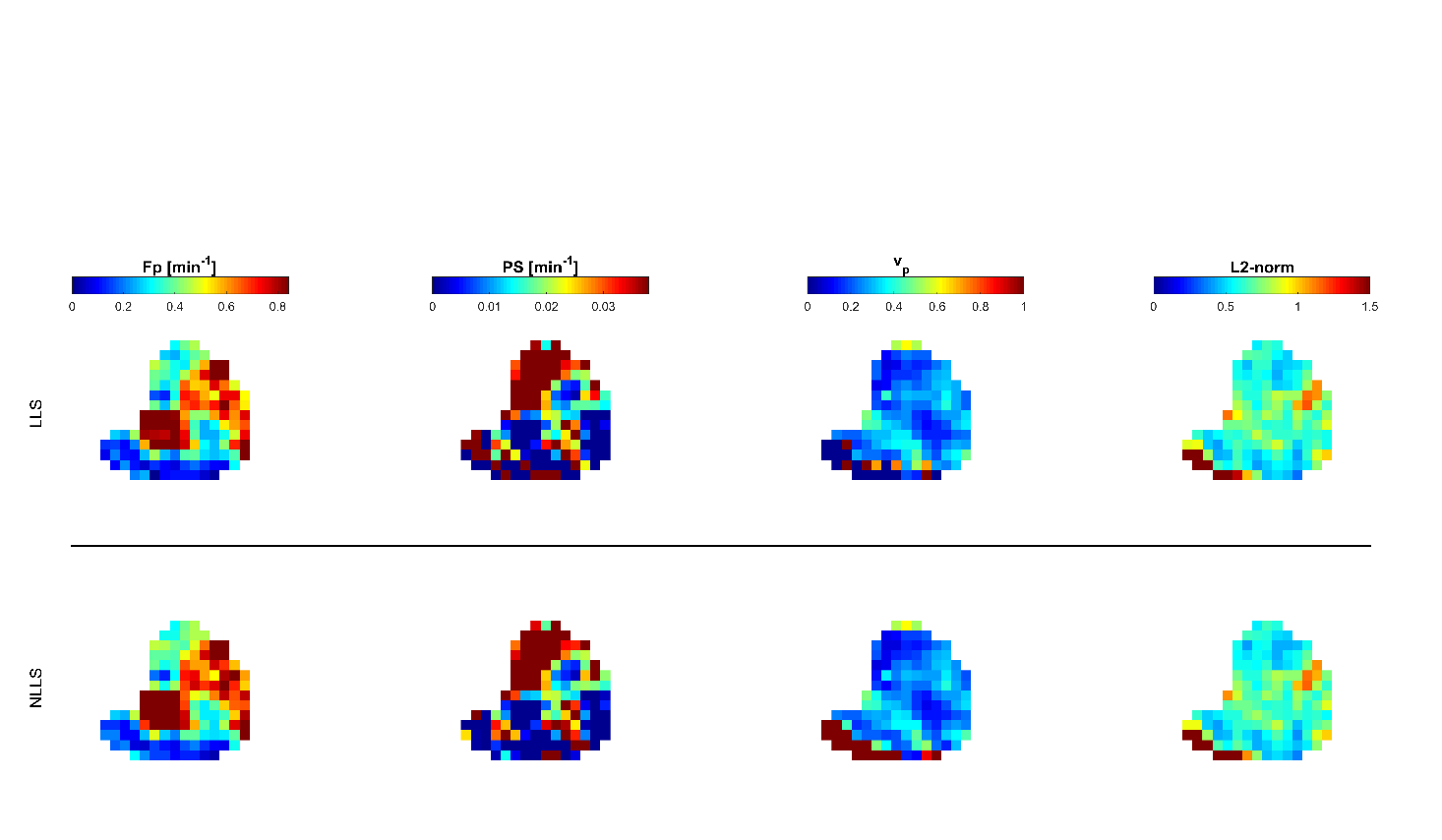


**Supporting Figure S3e:** Centre slice through tumour comparing estimated haemodynamic maps and goodness-of-fit using both NLLS and LLS (Patient 5).


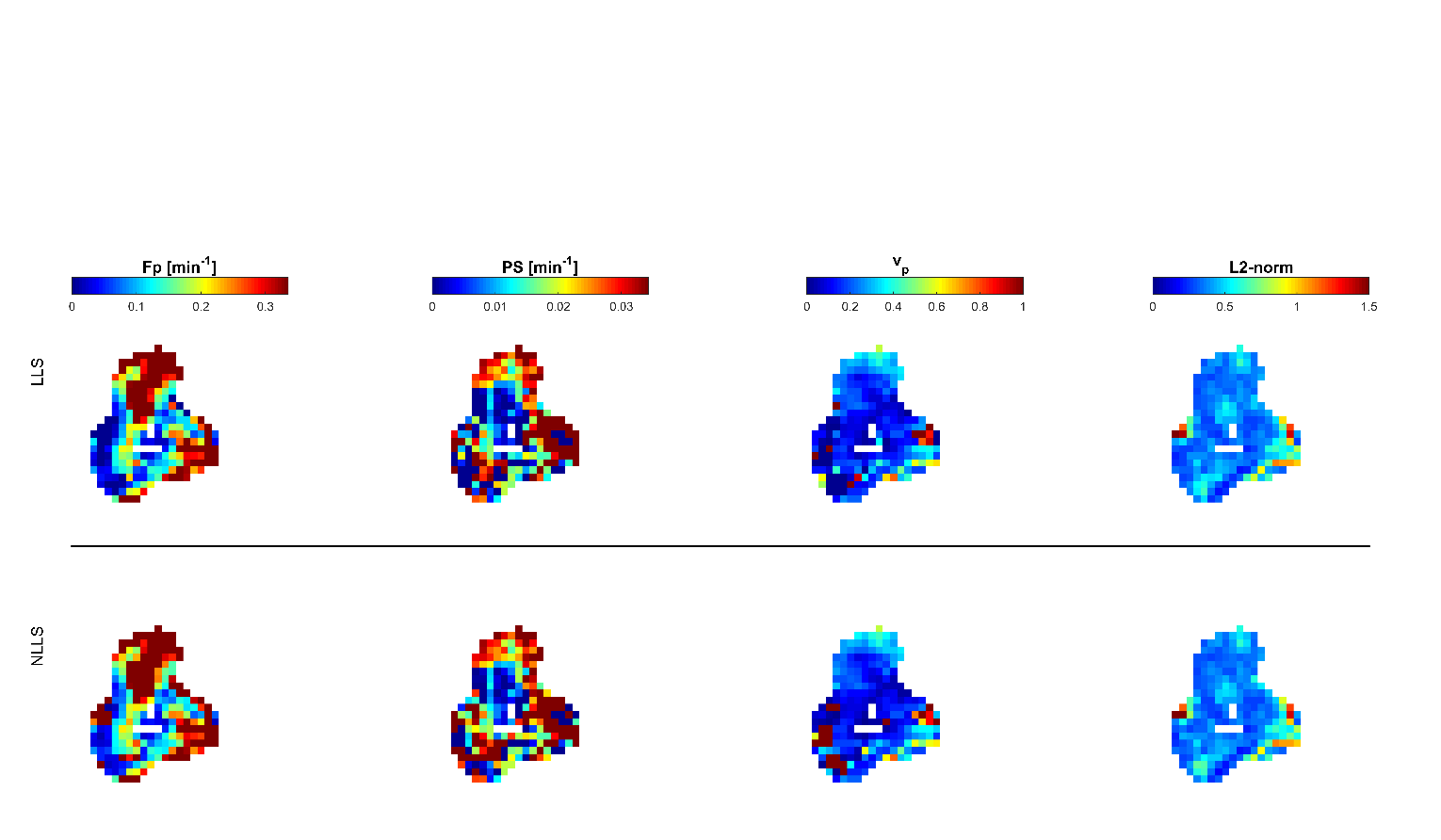


**Supporting Figure S3f:** Centre slice through tumour comparing estimated haemodynamic maps and goodness-of-fit using both NLLS and LLS (Patient 6).


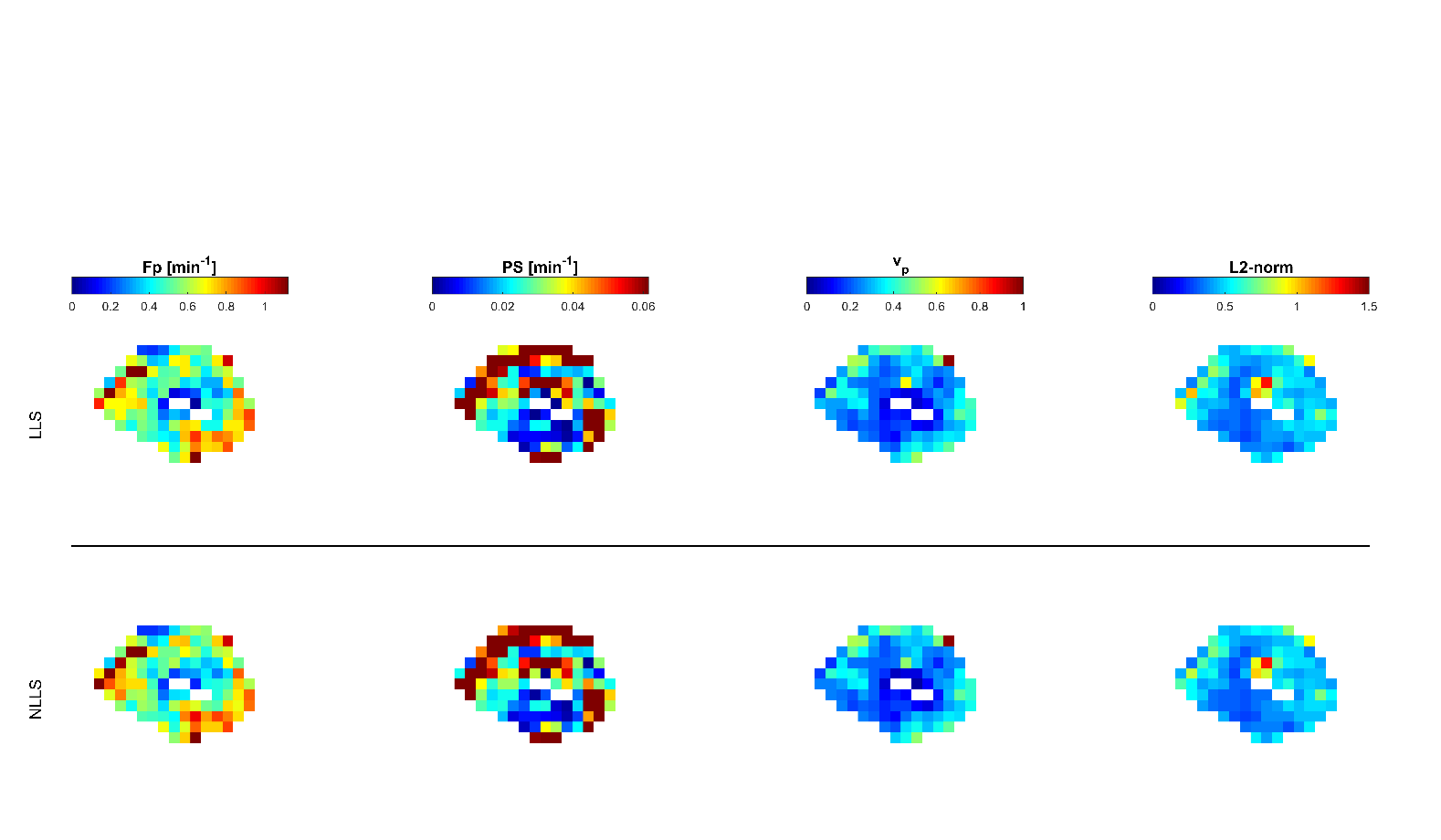


**Supporting Figure S3g:** Centre slice through tumour comparing estimated haemodynamic maps and goodness-of-fit using both NLLS and LLS (Patient 7).


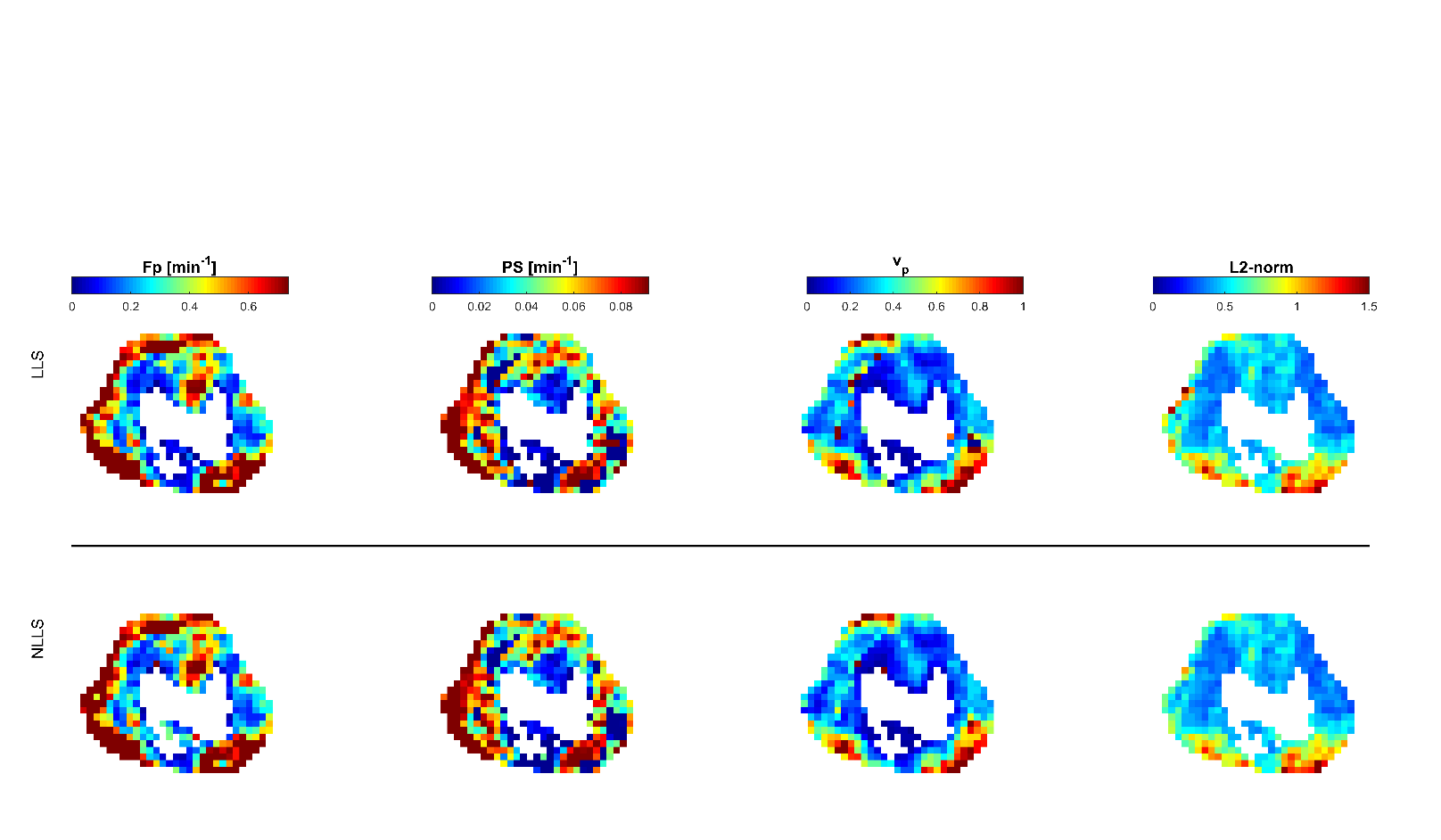


**Supporting Figure S3h:** Centre slice through tumour comparing estimated haemodynamic maps and goodness-of-fit using both NLLS and LLS (Patient 8).


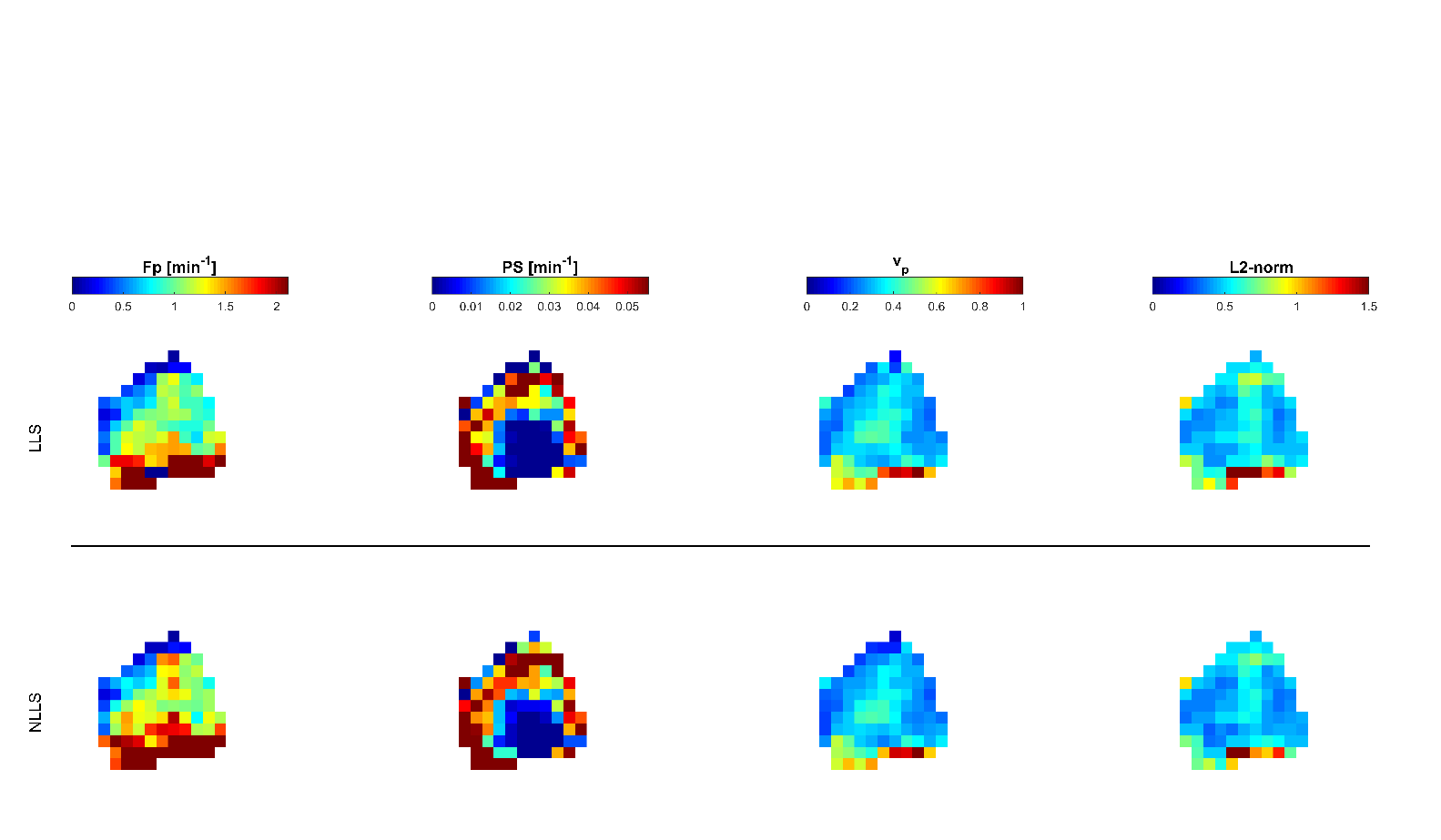


**Supporting Figure S3i:** Centre slice through tumour comparing estimated haemodynamic maps and goodness-of-fit using both NLLS and LLS (Patient 9).


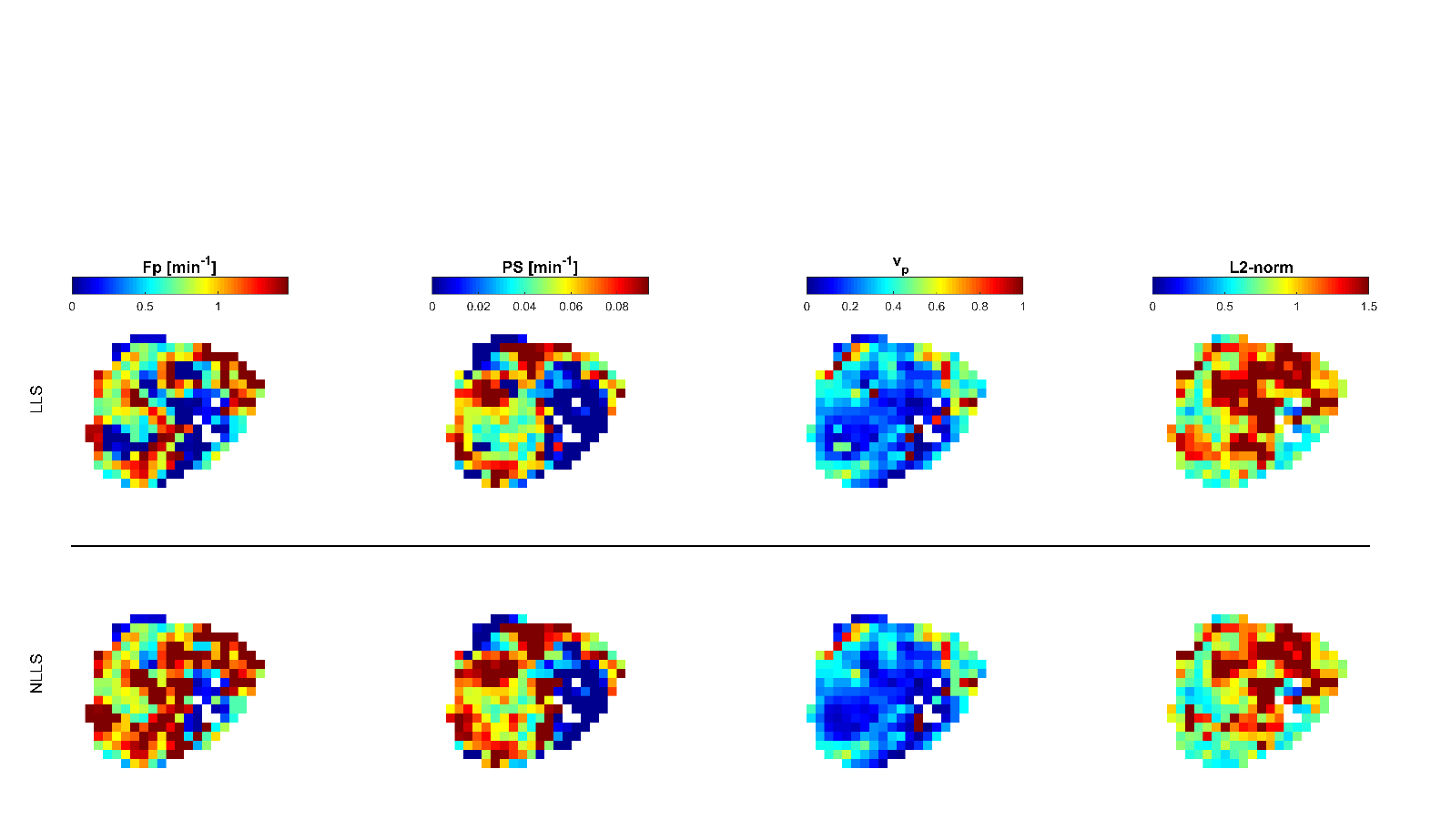


**Supporting Figure S3j:** Centre slice through tumour comparing estimated haemodynamic maps and goodness-of-fit using both NLLS and LLS (Patient 10).


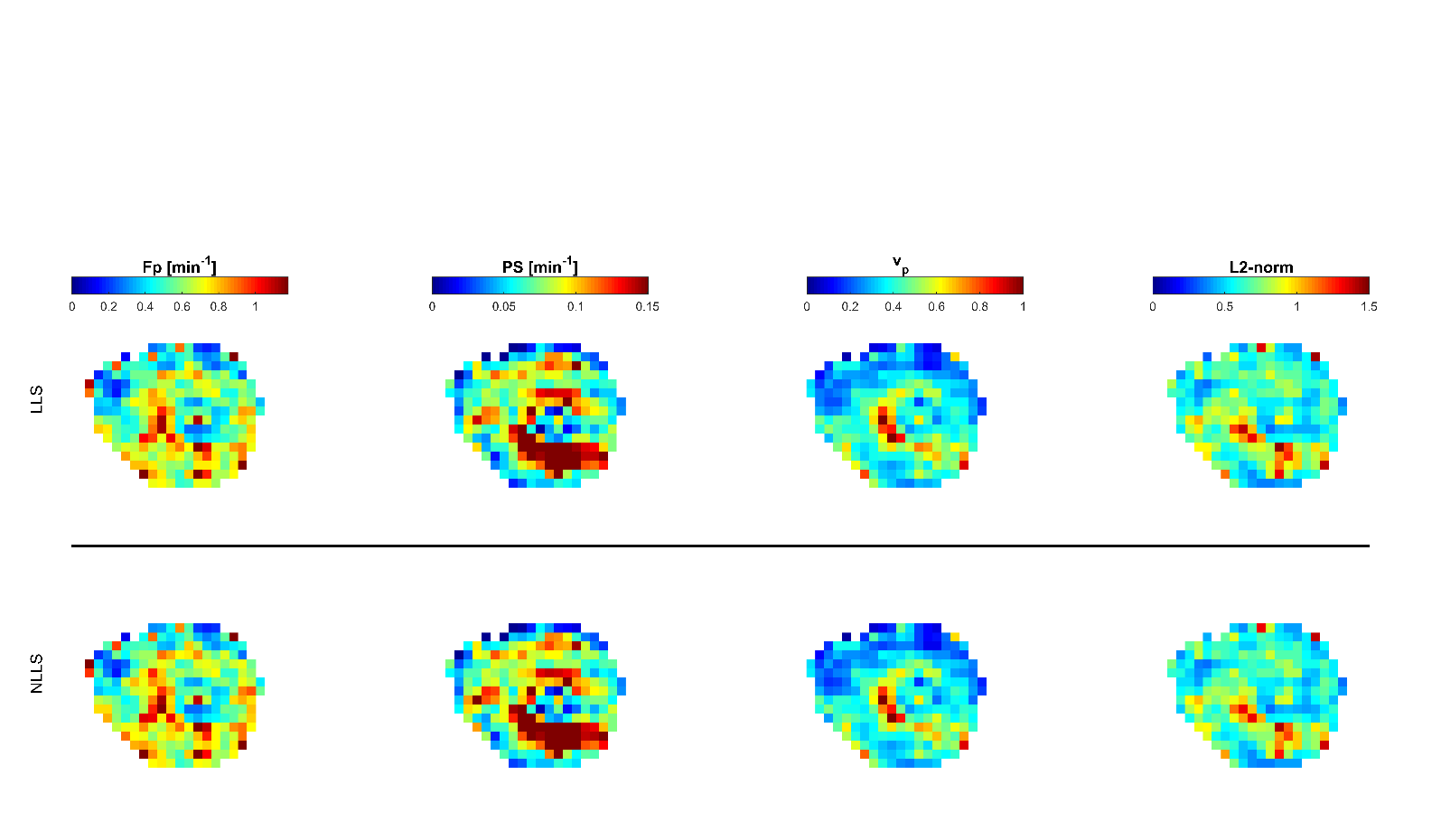


**Supporting Figure S3k:** Centre slice through tumour comparing estimated haemodynamic maps and goodness-of-fit using both NLLS and LLS (Patient 11).


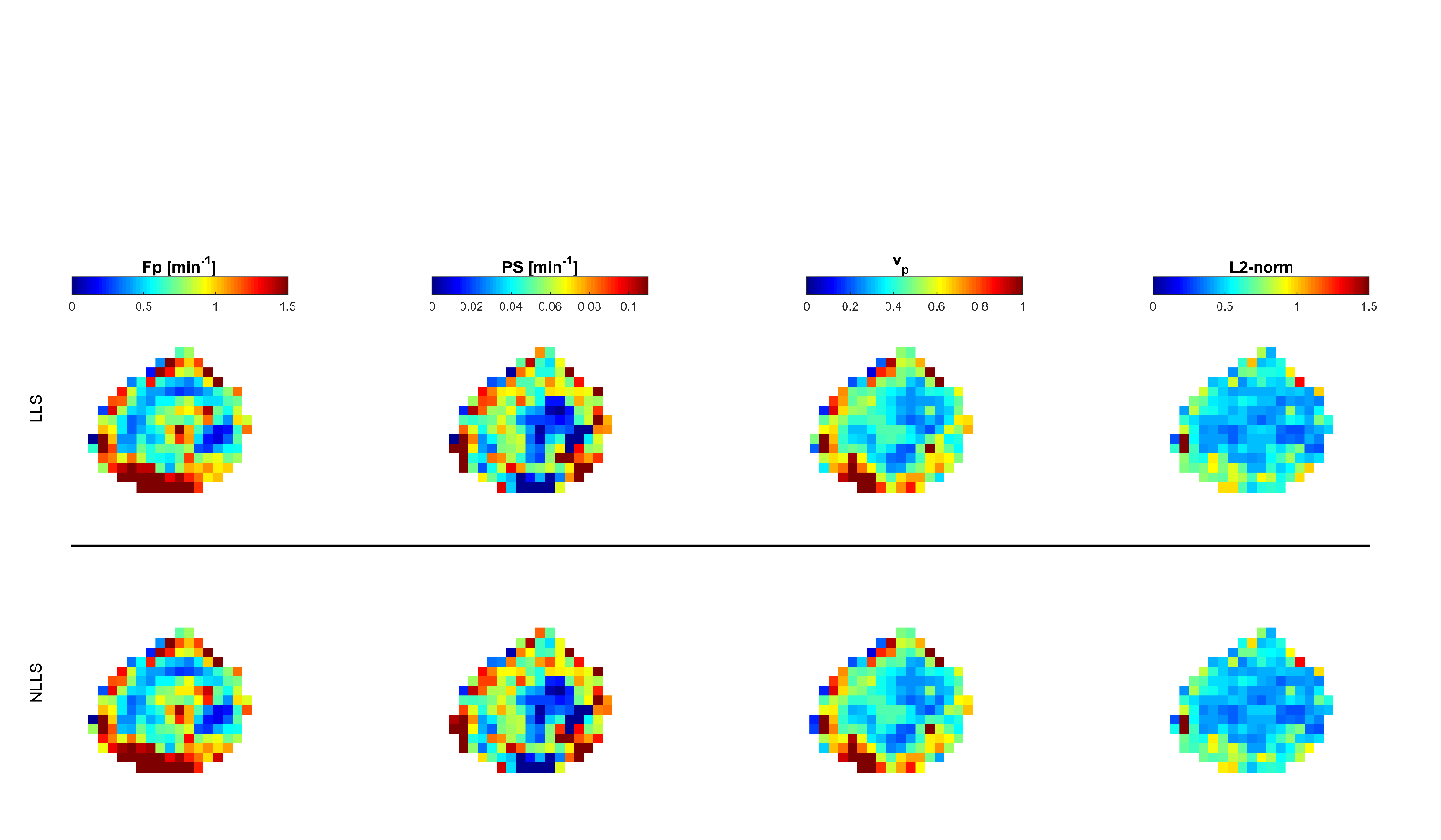


**Supporting Figure S3l:** Centre slice through tumour comparing estimated haemodynamic maps and goodness-of-fit using both NLLS and LLS (Patient 12).


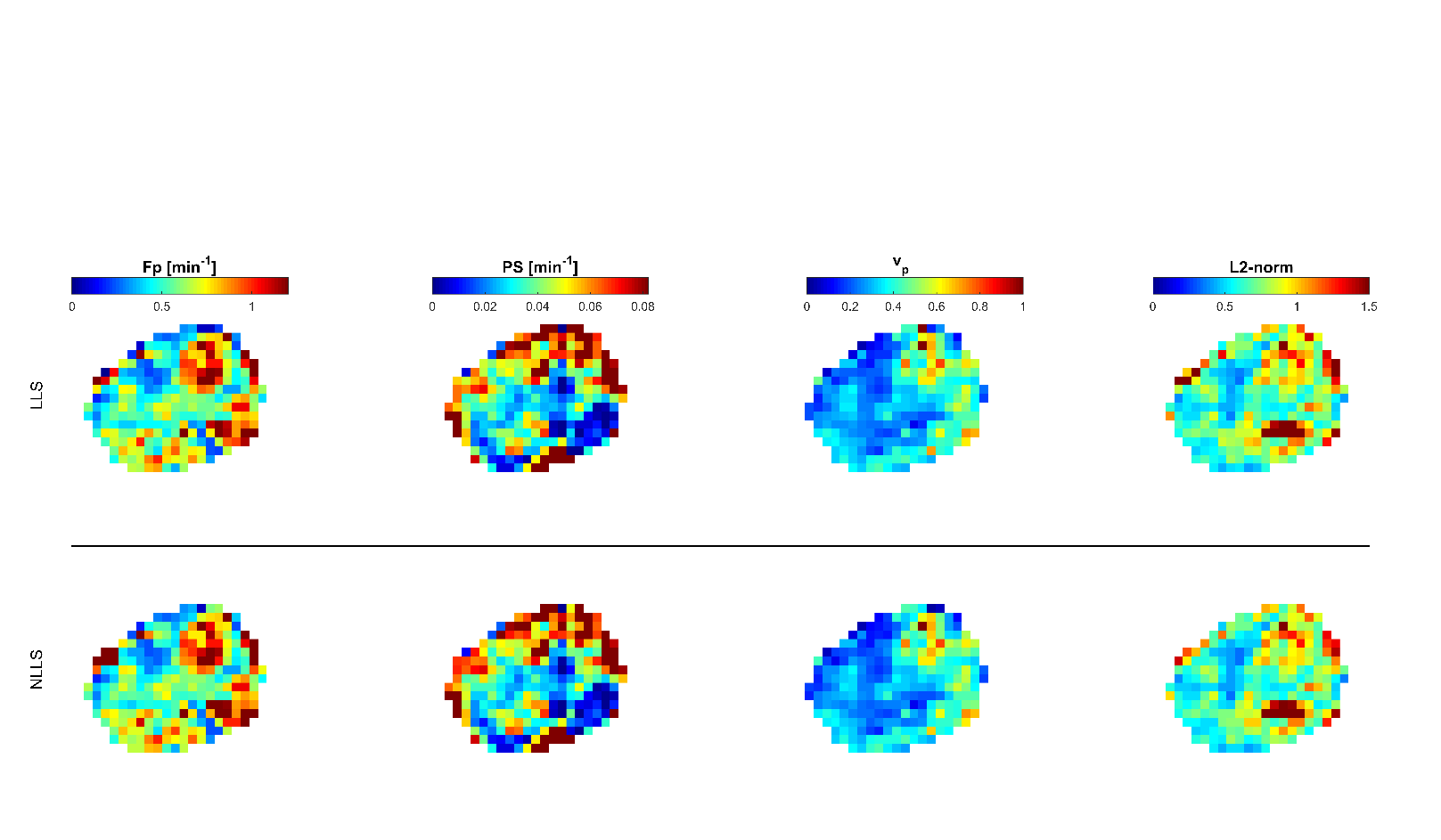


**Supporting Figure S3m:** Centre slice through tumour comparing estimated haemodynamic maps and goodness-of-fit using both NLLS and LLS (Patient 13).


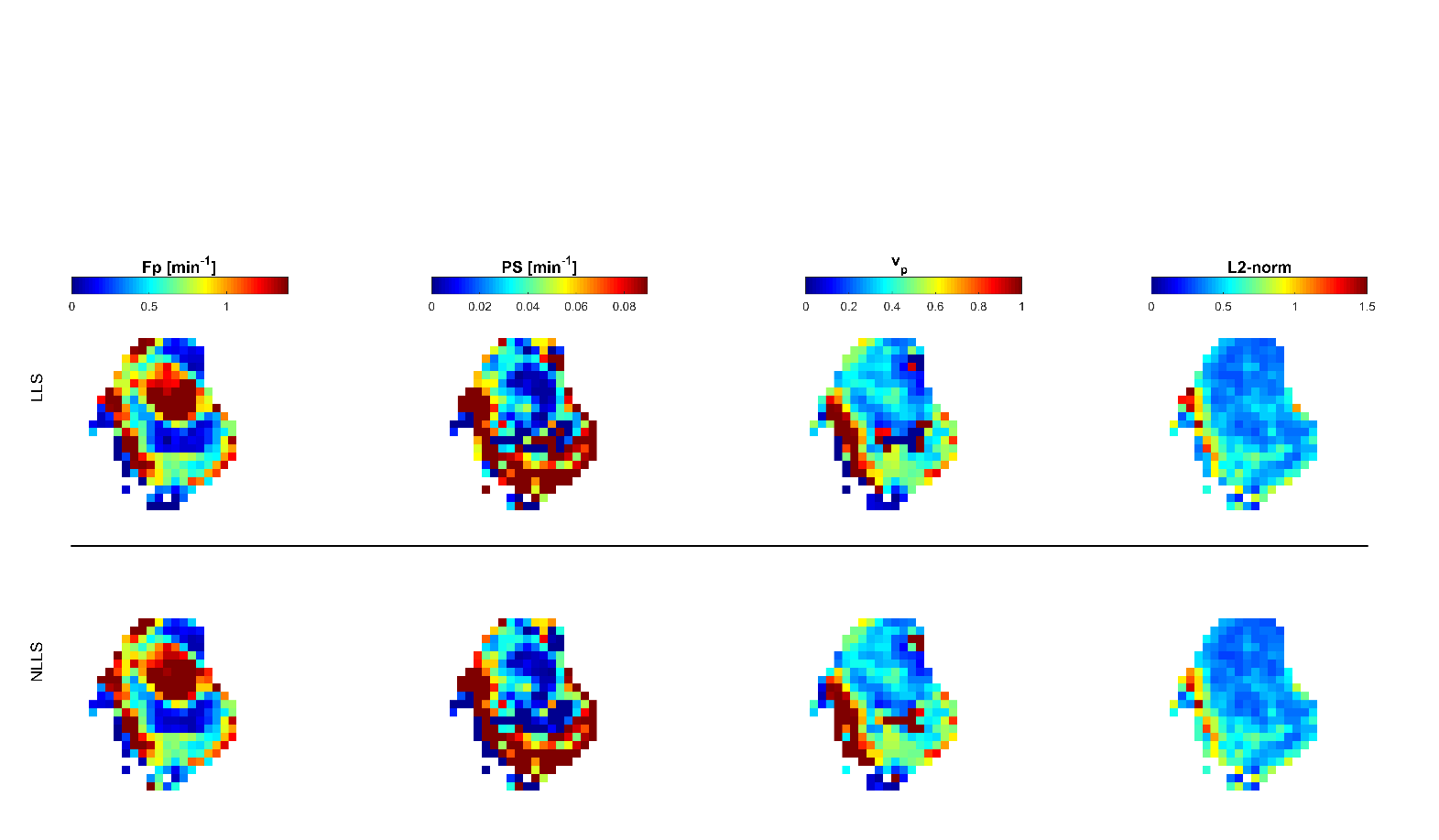


**Supporting Figure S3n:** Centre slice through tumour comparing estimated haemodynamic maps and goodness-of-fit using both NLLS and LLS (Patient 14).

**
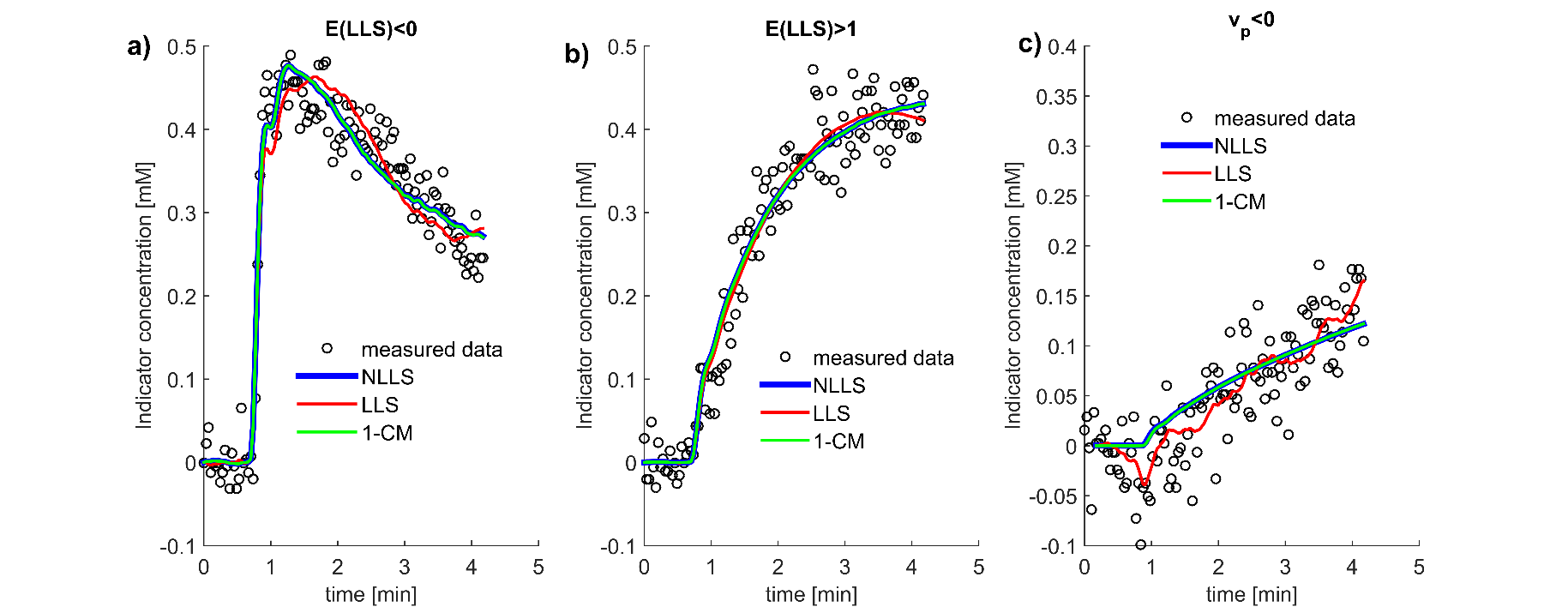
Supporting Figure S4:** Example curves excluded from the comparison of NLLS and LLS **a)** Typical data excluded when E(LLS)<0. **b)** Typical data excluded when E(LLS)>1. **c)** Typical data excluded when v_p_(LLS)<0. For comparison we included also included the fit of the one compartment model ($C\left( t \right)=c_{a}(t)\bigotimes(F_{p}e^{-{t{\cdot F}_{p}}/{v_{p}}}$)).
